# Causal pathways in lymphoid leukemia: the gut microbiota, immune cells, and serum metabolites

**DOI:** 10.3389/fimmu.2024.1437869

**Published:** 2024-09-16

**Authors:** Xin Zhuang, Qingning Yin, Rong Yang, Xiaoying Man, Ruochen Wang, Hui Geng, Yifen Shi

**Affiliations:** ^1^ Department of Hematology, The First Affiliated Hospital of Wenzhou Medical University, Wenzhou, China; ^2^ Department of Hematology, Qinghai Province Women and Children’s Hospital, Xining, Qinghai, China; ^3^ Department of Hematology, Affiliated Hospital of Qinghai University, Xining, Qinghai, China; ^4^ Department of Vice President, Qinghai Province Women and Children’s Hospital, Xining, Qinghai, China; ^5^ Zhejiang Provincial Clinical Research Center For Hematological Disorders, Wenzhou, China

**Keywords:** gut microbiota, immune cells, lymphocytic leukemia, Mendelian randomization analysis, serum metabolites

## Abstract

**Background:**

We employed Mendelian randomization (MR) to investigate the causal relationship between the gut microbiota and lymphoid leukemia, further exploring the causal relationships among immune cells, lymphoid leukemia, and potential metabolic mediators.

**Methods:**

We utilized data from the largest genome-wide association studies to date, encompassing 418 species of gut microbiota, 713 types of immune cells, and 1,400 serum metabolites as exposures. Summary statistics for lymphoid leukemia, acute lymphocytic leukemia (ALL), and chronic lymphocytic leukemia (CLL) were obtained from the FinnGen database. We performed bidirectional Mendelian analyses to explore the causal relationships among the gut microbiota, immune cells, serum metabolites, and lymphoid leukemia. Additionally, we conducted a two-step mediation analysis to identify potential intermediary metabolites between immune cells and lymphoid leukemia.

**Results:**

Several gut microbiota were found to have causal relationships with lymphoid leukemia, ALL, and CLL, particularly within the *Firmicutes* and *Bacteroidetes phyla*. In the two-step MR analysis, various steroid hormone metabolites (such as DHEAS, pregnenolone sulfateprogestogen derivatives, and androstenediol-related compounds) were identified as potential intermediary metabolites between lymphoid leukemia and immune cells. In ALL, the causal relationship between 1-palmitoyl-2-docosahexaenoyl-GPE (16:0/22:6) and ALL was mediated by CD62L-plasmacytoid DC%DC (mediated proportion=-2.84%, *P*=0.020). In CLL, the causal relationship between N6,n6,n6-trimethyllysine and CLL was mediated by HLA DR+ CD8br AC (mediated proportion=4.07%, *P*=0.021).

**Conclusion:**

This MR study provides evidence supporting specific causal relationships between the gut microbiota and lymphoid leukemia, as well as between certain immune cells and lymphoid leukemia with potential intermediary metabolites.

## 1 Introduction

Lymphocytic leukemia, a hematologic malignancy, arises from progenitor cells within the B or T lymphocyte lineages. It manifests clinically with symptoms such as fever, bleeding, progressive anemia, and bone and joint pain. The acute variant predominantly affects children, comprising 80% of acute leukemia cases in this group (1), whereas the chronic form is more common in middle-aged and elderly individuals. Annually, more than 3,000 new cases are diagnosed (2), contributing to approximately 25% of pediatric cancer-related fatalities (3). Over recent decades, the incidence of Acute Lymphocytic leukemia (ALL) across all racial groups in the USA has increased annually by about 1%, indicating that risk factors may be increasingly prevalent (4). Despite thorough investigation of these risk factors, the precise etiology of lymphocytic leukemia has not been determined. Consequently, clarifying the molecular mechanisms underlying its onset and progression is crucial, highlighting the need to identify molecular biomarkers that can signal relapse and metastasis.

Studies indicate that the maturation of the gut microbiota may be delayed in children with ALL, characterized by a consistent deficiency of bacterial groups that produce short-chain fatty acids. This deficiency could potentially promote immune dysregulation and increase the risk of transformation of pre-leukemic clones in response to common infectious triggers (5). Additionally, Bifidobacteria have been shown to reduce tumor cell proliferation by inhibiting growth factor signaling and inducing mitochondrial-mediated apoptosis (6). However, further research involving human subjects, particularly pediatric populations, is essential to deepen our understanding of the relationship between the gut microbiome and lymphoid leukemia.

Immune cells combat tumor growth by recognizing and lysing tumor cells (7). In cases of lymphocytic leukemia, leukemic cells modify the phenotype and functionality of immune cells to evade immune surveillance (8, 9). Research has revealed that interleukin 10 (IL-10) deficiency in pediatric B-ALL indirectly suppresses B lymphocyte production and exacerbates B cell DNA damage associated with six pro-inflammatory cytokines (10). Moreover, elevated levels of the chemokine PARC have been identified in pediatric acute lymphocytic leukemia, suggesting that serum PARC levels could serve as a novel biomarker for leukemia, indicative of the interactions between tumor cells and host cells (11). Nonetheless, observational studies are prone to measurement errors, uncontrollable confounding factors, and reverse causality, which may skew results. Thus, employing Mendelian randomization is crucial to mitigate these biases and confirm the causal relationship between immune cells and lymphocytic leukemia.

In lymphocytic leukemia, leukemic cells undergo metabolic reprogramming driven by genetic mutations, facilitating their growth and development (12). Metabolomic analysis of patient plasma and urine indicates significant changes in metabolites pre- and post-treatment, suggesting new avenues for identifying prognostic biomarkers and underscoring the potential therapeutic benefits of targeting metabolic pathways in this condition (13). Studies have demonstrated that amino acid metabolites serve as activators of immune surveillance and as carriers for drugs targeting T-cell acute lymphocytic leukaemia (14). Consequently, the potential causal link between serum metabolites and lymphoid leukemia merits further investigation, particularly regarding the role of serum metabolites as mediators between immune cells and lymphocytic leukemia.

Mendelian Randomization (MR) utilizes genetic variation to establish the association between exposure factors and diseases in observational studies, thereby enhancing the causal inference between risk factors and outcomes. This method effectively reduces the impact of unmeasured errors and confounding factors by adhering to genetic principles, and mitigates the bias caused by reverse causality (15). MR has been widely applied in cancer research, providing valuable insights into the etiology of various cancers. For instance, a study summarized the relationship between 25-hydroxyvitamin D in serum and the risk of tumors in different systems, identifying four studies on the association between serum 25-hydroxyvitamin D and cancer mortality (16). Kim et al. analyzed 14 genetic predictive tools for micronutrient levels and applied two-sample MR to estimate their causal effects on 22 cancer outcomes (17). Increasing evidence also emphasizes the importance of using genetic data related to gut microbiota, immune cells, and metabolites in clinical research. For example, a two-sample MR analysis was conducted to assess the causal effect of gut bacteria on the risk of five different types of cancer (18). In this study, we applied two-sample MR to infer the causal relationship between gut microbiota, immune cells, metabolites, and lymphocytic leukemia. Further, we combined mediator Mendelian analysis to assess the mediating effects of serum metabolites in the interaction between immune cells and lymphocytic leukemia.

## 2 Materials and methods

### 2.1 Ethical approval and consent to participate

Based on publicly available data, this study received ethical approval and consent for participation. Each constituent study within the Genome Wide Association Studies (GWAS) was approved by the respective institutional review board, with informed consent obtained from participants or their caregivers, legal guardians, or authorized proxies.

### 2.2 Research design

**Figure 1:**
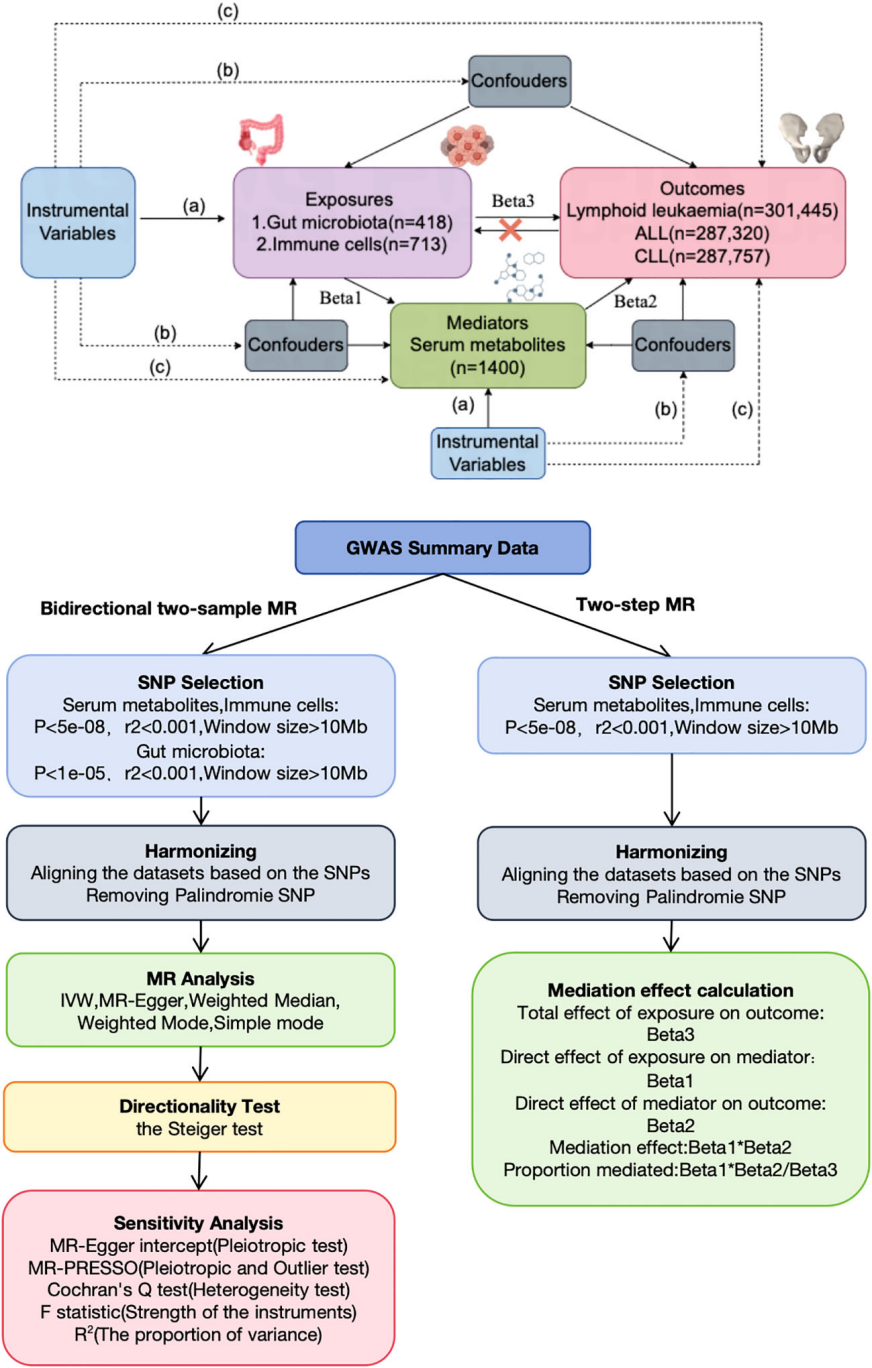
Assumptions and design of the bidirectional and mediation MR analyses. Firstly, a two-sample bidirectional MR was performed to investigate the causal relationships between Gut microbiota,Immune cells,Serum metabolites (exposure) and Lymphoid leukaemia,ALL,CLL (outcome). Secondly, Serum metabolites (mediator) were selected for subsequent mediation analyses. Finally, a two-step MR analysis was conducted to detect potential mediating metabolites (Step1, the effect of Immune cells on Serum metabolites; Step2, the effect of Serum metabolites on Lymphoid leukaemia,ALL,CLL).

This study, based on the STROBE-MR statement (19), investigated the bidirectional causal relationships among serum metabolites, immune cells, gut microbiota, and lymphoid leukemia, including ALL and CLL, using two-sample MR. MR employs genetic variations as proxies for risk factors, thereby serving as an effective tool for causal inference. For an instrumental variable (IV) in MR to be valid, it must satisfy three critical assumptions: (1) the genetic variation is directly associated with the exposure; (2) it is unassociated with any potential confounders of the exposure and outcome; (3) it affects the outcome solely through the exposure and not through alternative pathways. Our analyses were conducted with approval from relevant institutional review boards, and informed consent was provided by all participants. Additionally, we utilized a two-step MR approach to examine the mediating role of serum metabolites on the effects of immune cells on lymphoid leukemia, including ALL and CLL (**Figure 1**).

### 2.3 Data source

Plasma metabolite data were derived from a comprehensive series of Genome-Wide Association Studies (GWAS) within the Canadian Longitudinal Study on Aging (CLSA) cohort, analyzing 1,091 metabolites and 309 metabolite ratios in 8,299 participants. The CLSA includes participants from all ten provinces of Canada and collects comprehensive data and biospecimens. Among those who provided blood, approximately 60 mL of non-fasting blood was collected into six types of tubes, yielding ten fraction types including serum, four types of plasma (citrate, platelet poor citrate, heparin, and ethylenediaminetetraacetic acid (EDTA)), buffy coat, two types of peripheral blood mononuclear cells (with and without cell preservative), and three types of whole blood (acid citrate dextrose, EDTA), including dried blood spots (baseline only). Biospecimen collection and processing were conducted in dedicated laboratories at each DCS. Blood samples were processed within 2 hours of collection and were temporarily stored at −80°C before being shipped weekly in cryoshippers to the CLSA Biorepository and Bioanalysis Centre (BBC) for long-term storage in cryofreezers (−190°C) (20). This metabolomics study focused on 8,299 unrelated European participants in the CLSA who had undergone whole-genome genotyping and had circulating plasma metabolites measured. The study focused on individuals of European descent to reduce potential biases due to population stratification. Using kinship-based inference from the KING package (v2.2.5), 203 European individuals with first and second-degree relatives were removed. Whole-genome genotyping was completed using the Affymetrix Axiom Genotyping Platform, followed by imputation using the Trans-Omics for Precision Medicine (TOPMed) protocol and determination of genetic ancestry by the CLSA group. Subsequently, we removed low-quality imputed genetic variants by retaining only those SNPs with a minor allele frequency (MAF) greater than 0.1, imputation quality score > 0.3, and missing rate < 0.1, resulting in approximately 15.4 million SNPs for GWAS testing. Metabolon, Inc. (Durham, North Carolina, USA) quantified the levels of metabolites in plasma samples using an Ultrahigh Performance Liquid Chromatography-Tandem Mass Spectroscopy (UPLC-MS/MS) platform. Metabolomics data underwent rigorous quality control and management to ensure accurate and consistent identification of true chemical entities and to remove those representing systemic artifacts, misalignments, and background noise. We then used the batch-normalized levels of metabolites and retained only those metabolites with missing measurements in fewer than 50% of the samples. For GWAS, metabolite levels were then natural log-transformed, trimmed to remove outliers that are 3 standard deviations away, and then standardized to have a mean of 0 and a standard deviation of 1. For metabolite ratios, we first identified 309 pairs of metabolites that share enzymes or transporters using the Human Metabolome Database (HMDB). The metabolite ratios for each pair were then calculated by dividing the batch-normalized measurement of one metabolite by that of the other in the same individual. The metabolite ratios were then trimmed (retaining those within 3 standard deviations), and inverse-rank normal transformed (21). Summary statistics for 731 immune phenotypes, including Absolute Cell (AC) counts (n = 118), Median Fluorescence Intensity (MFI) (n = 389), Morphological Parameters (MP) (n = 32), and Relative Cell (RC) counts (n = 192), are available from the GWAS Catalog (entries GCST0001391 to GCST0002121) (22). These parameters encompass various cell types such as B cells, CDCs, T cells at different maturation stages, monocytes, bone marrow cells, TBNK (T cells, B cells, Natural Killer cells), and Treg panels, with MP focusing on CDC and TBNK. The initial GWAS of immune traits included data from 3,757 European individuals, using about 22 million SNPs characterized by high-density array genotyping from a Sardinian sequence reference panel (23), with associations assessed post-adjustment for covariates like sex, age, and age squared. Additionally, 418 gut microbiota entries from the NHGRI-EBI GWAS Catalog were utilized (entries ebi-a-GCST90027857 to ebi-a-GCST90027857440).

Moreover, this study incorporated GWAS data on lymphoid leukemia (case=1,493, control=299,952), ALL (case=184, control=287,136), and CLL (case=624, control=287,133) from the FinnGen consortium (https://www.finngen.fi/en). These datasets, which excluded all cancer types, were sourced from public domains, thereby circumventing any ethical and copyright issues.

### 2.4 Genetic instrumental variable selection

A key element of MR studies is the use of single nucleotide polymorphisms (SNPs) as IVs to address confounding factors in observational research. Valid IVs are selected through rigorous criteria, with SNPs associated with serum metabolites and immune cells identified at a significance threshold of *P*<5×10^−8^. For gut microbiota exposure, a genome-wide significance threshold is set at 1×10^-5^. These SNPs serve as genetic instrumental variables. We also compute the F-statistic for each genetic instrument, where(R^2^(n-2)/(1-r^2^))measures the instrument’s strength, R^2^ is the proportion of variance explained, and n is the effective sample size in the GWAS. A threshold F-value >10 indicates robust estimates in MR analysis. To minimize bias due to linkage disequilibrium (LD), we cluster SNPs within a ±10,000 kb range using an LD threshold of r^2^ < 0.001, based on the 1000 Genomes European reference panel, including only SNPs with a minor allele frequency > 0.01. We ensure SNP effects on specific outcomes and exposures are allele-specific. Palindromic SNPs are excluded from the analysis.

### 2.5 Bidirectional two-sample and mediation analysis

We conducted bidirectional two-sample analyses to evaluate causal relationships among serum metabolites, lymphoid leukemia (ALL, CLL), immune cells, and gut microbiota. For multiple IVs, the inverse variance weighted (IVW) method with multiplicative random effects is optimal for estimating causal effects and addressing heterogeneity (24). Therefore, we selected the IVW method with multiplicative random effects as our primary MR analysis technique. For single IV exposures, we applied the Wald ratio method to estimate causality. Additionally, we utilized two-step MR for mediation analysis to determine if serum metabolites mediate causal pathways from immune cells to lymphoma outcomes. We calculated the mediation proportion of serum metabolites as the indirect effect divided by the total effect (β1×β2/β3), where β1 measures the impact of immune cells on serum metabolites, β2 measures the impact of serum metabolites on the outcome, and β3 measures the impact of immune cells on the outcome. The 95% confidence interval is calculated using the delta method.

### 2.6 Sensitivity analysis

We further evaluated the robustness of significant and potential causal relationships using various statistical methods: MR Egger regression (25), Weighted Median (26), Weighted Mode (27), Simple Mode, and MR Pleiotropy Residual Sum and Outlier (MR-PRESSO) (28). These methods are crucial as they enable the detection of violations of MR assumptions under varied assumptions (29). Additionally, we conducted further sensitivity analyses, including the calculation of Cochran’s Q statistic to evaluate the heterogeneity of causal inference (30), and leave-one-out analysis to determine the influence of specific variables on the estimates of causal effects (31). We also applied the MR Steiger directionality test to ascertain the direction of causal relationships between exposures and outcomes. When the Steiger test revealed stronger associations between certain genetic IVs and outcomes, we excluded these variants and performed a reanalysis (32).

### 2.7 Statistical analyses

MR analyses were performed using R software (version 4.3.3, http://www.r-project.org) and the TwoSampleMR package. The MR-Pleiotropy Residual Sum and Outlier analyses were executed using the MR.raps R package. To evaluate all known phenotypes associated with our genetic tools, we utilized PhenoScanner.

## 3 Result

### 3.1 Instrument variables included in analysis

The following criteria were used to select the best IVs to enhance the authenticity and accuracy of the research conclusions. (1) When selecting serum metabolites and immune cells as exposures, to identify more stringent SNPs, we use a genome-wide significance threshold (*P*<5×10^-8^) to select IVs. When gut microbiota data is defined as exposure, the *P-value* threshold is relaxed to 1×10^-5^ to ensure an adequate number of SNPs are included in the analysis. When lymphoid leukemia, ALL, and CLL are chosen as outcomes, we use a genome-wide significance threshold (*P*<5×10^-8^) to select IVs. (2) Due to the potential bias caused by strong LD, we ensure there is no LD among the selected IVs. Data from the 1000 Genomes Project European samples are used as a reference panel to calculate LD between SNPs, and SNPs that meet the threshold (r^2^<0.001, kb=10,000) are retained for further analysis. (3) The F-statistics of the selected IVs reach a threshold of >10, ensuring that the causal estimates are free from weak instrument bias.

### 3.2 Causal effects of gut microbiota on lymphoid leukemia

**Figure 2:**
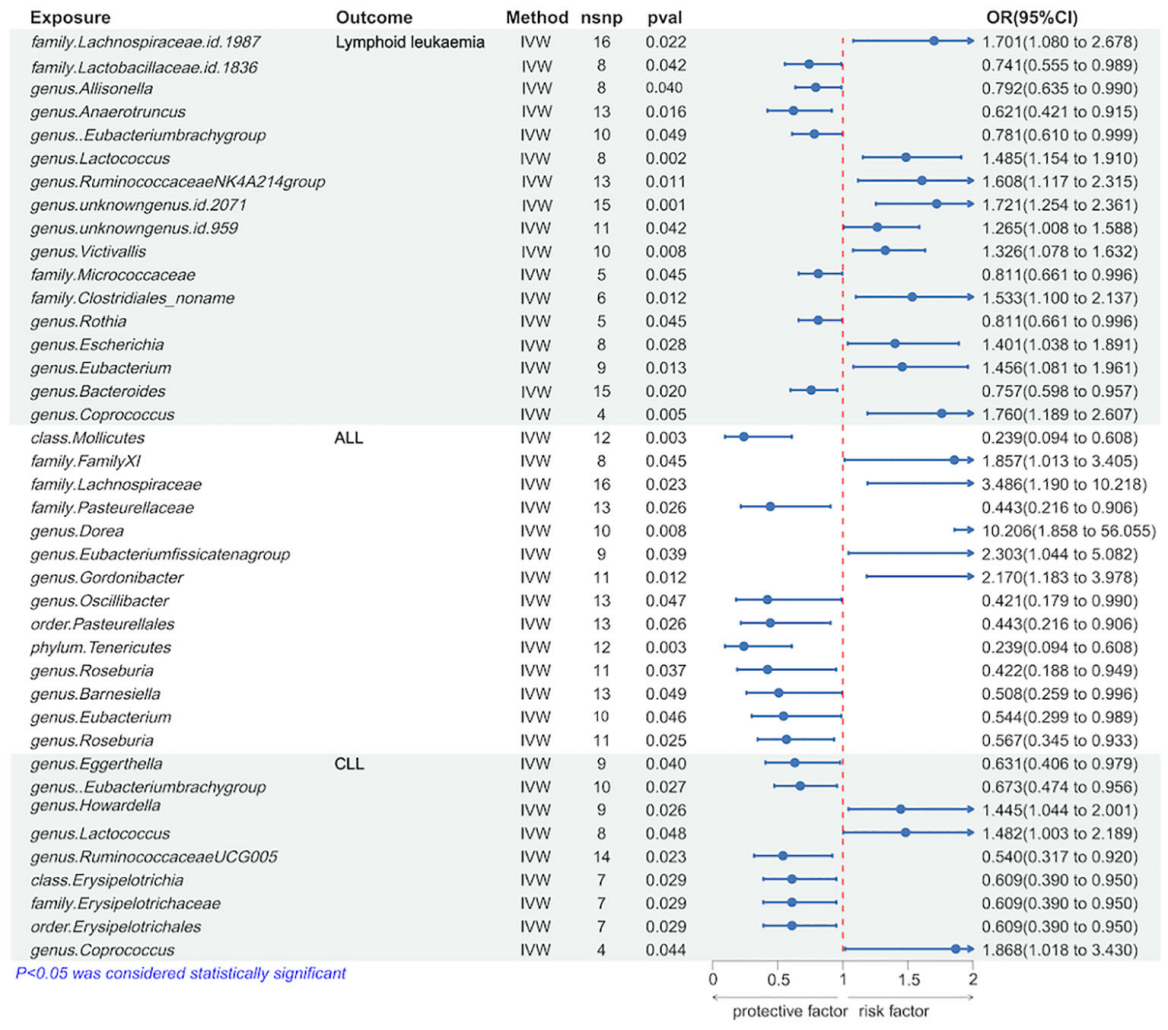
Causal effects of Lymphoid leukaemia on Gut Microbiota.

We utilized the IVW method to identify gut microbiota significantly causally associated with lymphoid leukemia, including ALL and CLL. Several types of gut microbiota were found to be significantly related to lymphoid leukemia, ALL, and CLL. Specifically, 17 types were significantly associated with lymphoid leukemia, 14 types with ALL, and 9 types with CLL. Further analysis was conducted on exposures and outcomes with SNP counts of three or more. A higher genetically predicted level of *genus.Coprococcus* was associated with an increased risk of lymphoid leukemia (OR[95%CI]=1.760[1.189-2.607], *P*=4.8e−03), while a higher level of genus.Anaerotruncus was associated with a decreased risk of lymphoid leukaemia (OR[95% CI]=0.621[0.421-0.915], *P* =1.6e−02). Additionally, a higher genetically predicted level of *genus.Dorea* was linked with an increased risk of ALL (OR[95%CI]=10.206[1.858-56.055],*P*=7.5e−03), and a higher level of *family.Pasteurellaceae* was linked with a decreased risk of ALL (OR[95% CI]=0.443[0.216-0.906], *P*=2.6e−02). Similarly, a higher genetically predicted level of *genus.Coprococcus* was associated with an increased risk of CLL (OR[95% CI]=1.868[1.018-3.430], *P*=4.4e−02), and a higher level of *genus.Ruminococcaceae UCG005* was associated with a decreased risk of CLL (OR[95%CI] =0.540[0.317-0.920], *P*=2.3e−02) (**Figure 2** and **Supplementary Figure 4S1**).

Further analyses using MR Egger, weighted median, simple mode, and weighted mode methods have confirmed the genetic causality (**Supplementary Table 1**).

### 3.3 Causal effects of serum metabolites on lymphoid leukemia

**Figure 3:**
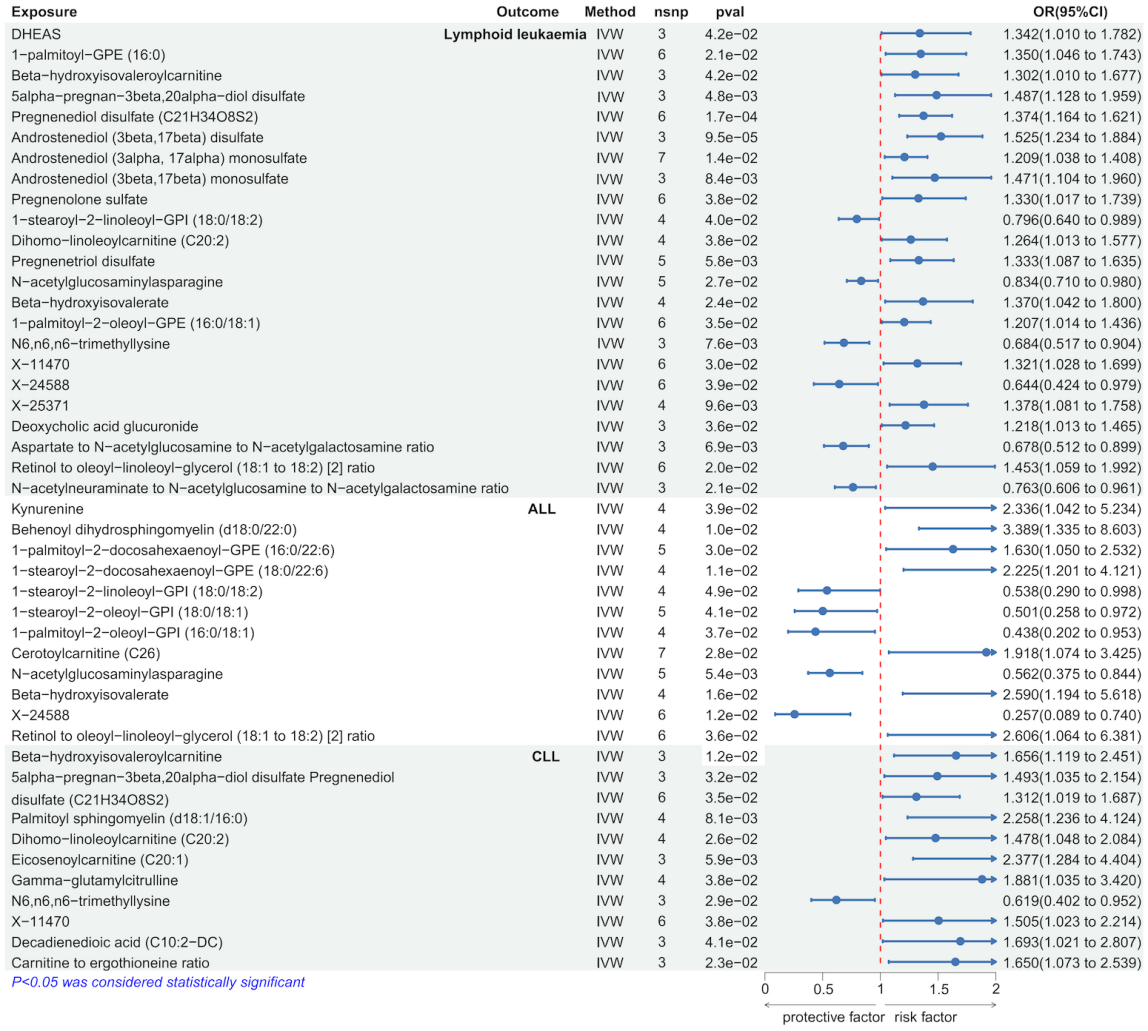
Causal effects of Lymphoid leukaemia on Serum metabolites.

We employed the IVW method to identify serum metabolites significantly causally associated with lymphoid leukemia, including ALL and CLL. Several serum metabolites were significantly associated with these lymphoid leukemia: 23 metabolites were significantly causally related to lymphoid leukemia, 12 to ALL, and 11 to CLL. Further analyses were conducted on exposures and outcomes with SNP counts of three or more. Higher genetically predicted levels of Androstenediol (3beta, 17beta) disulfate were associated with an increased risk of lymphoid leukemia (OR[95% CI]=1.525[1.234-1.884], *P* =9.5e−05), while higher levels of X-24588 were associated with a decreased risk of lymphoid leukemia (OR[95%CI]=0.644[0.424-0.979],*P*=3.9e−02). Higher levels of Behenoyl dihydrosphingomyelin (d18:0/22:0) were associated with an increased risk of ALL (OR[95%CI]=3.389[1.335-8.603], *P*=1.0e−02), and X-24588 was associated with a decreased risk of ALL (OR[95%CI]=0.257[0.089-0.740],*P*=1.2e−02). Higher levels of Eicosenoylcarnitine (C20:1) were associated with an increased risk of CLL (OR[95%CI]=2.377[1.284-4.404],*P*=5.9e−02), and higher levels of N6.n6, n6-trimethyllysine were associated with a decreased risk of CLL (OR[95%CI]=0.619[0.402-0.952], *P*=2.9e−02) (**Figure 3** and **Supplementary Figure 4S2**).

Further analyses using MR Egger, weighted median, simple mode, and weighted mode methods confirmed the genetic causality (**Supplementary Table 2**).

### 3.4 Causal effects of immune cells on lymphoid leukemia

**Figure 4:**
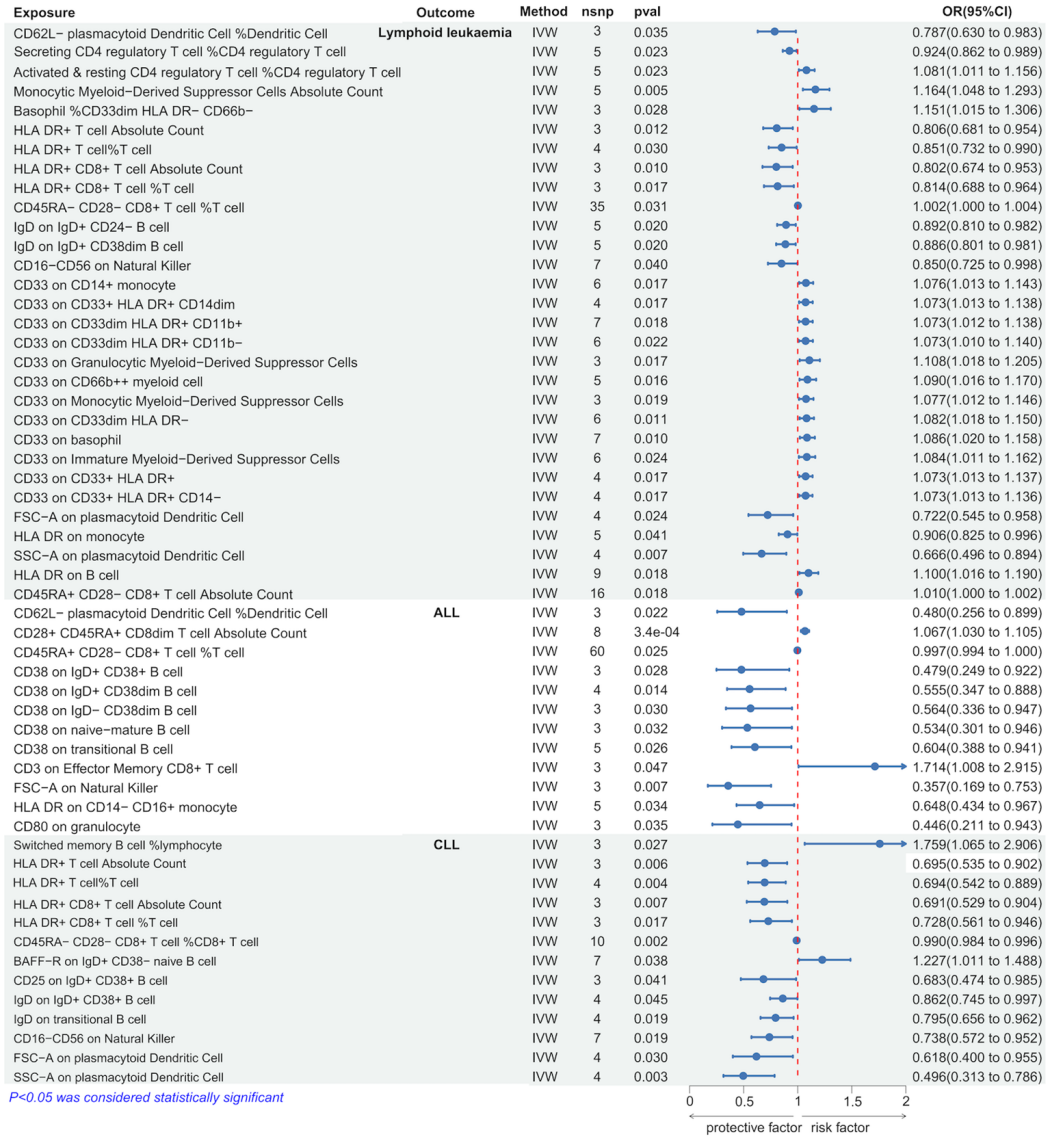
Causal effects of Lymphoid leukaemia on Immune Cells.

Using the IVW method, we discovered several immune cells significantly associated with lymphoid leukemia, ALL, and CLL. Specifically, 30 types of immune cells were significantly causally related to lymphoid leukemia, 12 types to ALL, and 13 types to CLL. Among these, higher genetically predicted levels of Monocytic Myeloid-Derived Suppressor Cells Absolute Count were associated with an increased risk of lymphoid leukemia (OR[95%CI]=1.164[1.048-1.293], *P*=4.5e−03), and higher SSC-A on plasmacytoid Dendritic Cells was associated with a decreased risk of lymphoid leukemia (OR[95%CI]=0.666 [0.496-0.894], *P*=6.7e−03). Higher CD3 on Effector Memory CD8+ T cells was associated with an increased risk of ALL (OR[95% CI]=1.714[1.008-2.915], *P*=4.7e−02), and higher CD80 on granulocytes was associated with a decreased risk of ALL (OR [95%CI]=0.446 [0.211-0.943], *P*=3.5e−02). Higher percentages of Switched memory B cells were linked with an increased risk of CLL (OR[95%CI]=1.759[1.065-2.906],*P*=2.7e−02), and higher SSC-A on plasmacytoid Dendritic Cells was associated with a decreased risk of CLL (OR[95%CI]=0.496[0.313-0.786],*P*=2.8e−03). (**Figure 4** and **Supplementary Figure 4S3**).

Further analysis using MR Egger, weighted median, simple mode, and weighted mode methods confirmed the genetic causality (**Supplementary Table 3**).

### 3.5 Sensitivity analysis

We assessed the heterogeneity and pleiotropy of gut microbiota, metabolites, and immune cells significantly associated with lymphoid leukemia, ALL, and CLL using the IVW model. P-values exceeding 0.05 indicate an absence of heterogeneity and pleiotropy among these variables (**Tables 1**–**3**). Sensitivity analyses employing the leave-one-out method for these variables confirmed the robustness of our findings, showing no significant influences from SNPs.

**Table 1:** Mendelian randomization analyses of the causal effects between gut microbiota and lymphocytic leukemia.

Outcome	Exposure	N SNP	Q statistic	P heterogeneity	P intercept	Steiger pval
Lymphoid leukemia	*family.Lachnospiraceae.id.1987*	16	21.473	0.122	0.606	0.574
	*family.Lactobacillaceae.id.1836*	8	3.797	0.803	0.775	0.429
	*genus.Allisonella*	8	7.670	0.363	0.607	0.245
	*genus.Anaerotruncus*	13	7.405	0.830	0.965	0.573
	*genus.Eubacteriumbrachygroup*	10	10.928	0.281	0.063	0.306
	*genus.Lactococcus*	8	6.221	0.514	0.588	0.270
	*genus.RuminococcaceaeNK4A214group*	13	9.275	0.679	0.884	0.535
	*genus.unknowngenus.id.2071*	15	11.490	0.647	0.941	0.462
	*genus.unknowngenus.id.959*	11	7.810	0.647	0.638	0.263
	*genus.Victivallis*	10	8.684	0.467	0.563	0.199
	*family.Micrococcaceae*	5	1.215	0.876	0.807	0.612
	*family.Clostridiales_noname*	6	3.978	0.553	0.923	0.621
	*genus.Rothia*	5	1.222	0.874	0.812	0.566
	*genus.Escherichia*	8	5.078	0.651	0.845	0.535
	*genus.Eubacterium*	9	5.099	0.747	0.392	0.496
	*genus.Bacteroides*	15	10.315	0.739	0.440	0.416
	*genus.Coprococcus*	4	1.668	0.644	0.482	0.701
ALL	*class.Mollicutes.id.3920*	12	9.120	0.611	0.962	0.531
	*family.FamilyXI.id.1936*	8	6.143	0.523	0.275	0.228
	*family.Lachnospiraceae.id.1987*	16	10.963	0.755	0.116	0.569
	*family.Pasteurellaceae.id.3689*	13	8.285	0.762	0.091	0.413
	*genus.Dorea.id.1997*	10	15.094	0.088	0.204	0.640
	*genus.Eubacteriumfissicatenagroup.id.14373*	9	11.347	0.183	0.668	0.327
	*genus.Gordonibacter.id.821*	11	11.316	0.333	0.132	0.197
	*genus.Oscillibacter.id.2063*	13	14.524	0.268	0.554	0.394
	*order.Pasteurellales.id.3688*	13	8.285	0.762	0.091	0.413
	*phylum.Tenericutes.id.3919*	12	9.120	0.611	0.962	0.531
	*genus.Roseburia*	11	11.616	0.312	0.718	0.442
	*genus.Barnesiella*	13	7.077	0.852	0.264	0.457
	*genus.Eubacterium*	10	5.194	0.817	0.590	0.495
	*genus.Roseburia*	11	7.117	0.714	0.419	0.455
CLL	*genus.Eggerthella*	9	9.177	0.328	0.614	0.395
	*genus.Eubacteriumbrachygroup*	10	9.249	0.415	0.165	0.306
	*genus.Howardella*	9	4.291	0.830	0.281	0.296
	*genus.Lactococcus*	8	4.730	0.693	0.348	0.269
	*genus.RuminococcaceaeUCG005*	14	14.568	0.335	0.866	0.552
	*class.Erysipelotrichia*	7	6.199	0.401	0.159	0.581
	*family.Erysipelotrichaceae*	7	6.198	0.401	0.159	0.581
	*order.Erysipelotrichales*	7	6.199	0.401	0.159	0.581
	*genus.Coprococcus*	4	2.354	0.502	0.461	0.701

NSNP refers to the number of SNPs used for analysis; the Q statistic is a statistical measure of heterogeneity, with a higher value indicating a more significant difference between study results.

**Table 2:** Mendelian randomization analyses of the causal effects between serum metabolites and Lymphoid leukaemia.

Outcome	Exposure	N snp	Q statistic	P heterogeneity	P intercept	Steiger pval
Lymphoid leukaemia	DHEAS	3	0.502	0.778	0.608	0.125
	1-palmitoyl-GPE (16:0)	6	5.474	0.361	0.440	0.058
	Beta-hydroxyisovaleroylcarnitine	3	0.494	0.781	0.720	0.007
	5alpha-pregnan-3beta,20alpha-diol disulfate	3	2.656	0.265	0.923	0.009
	Pregnenediol disulfate (C21H34O8S2)	6	0.853	0.974	0.852	5.69E-16
	Androstenediol (3beta,17beta) disulfate	3	0.354	0.838	0.751	3.70E-06
	Androstenediol (3alpha, 17alpha) monosulfate	7	9.603	0.142	0.761	1.99E-11
	Androstenediol (3beta,17beta) monosulfate	3	1.253	0.535	0.656	0.223
	Pregnenolone sulfate	6	1.419	0.922	0.520	0.105
	1-stearoyl-2-linoleoyl-GPI (18:0/18:2)	4	0.981	0.806	0.498	0.003
	Dihomo-linoleoylcarnitine (C20:2)	4	0.956	0.812	0.576	0.0003
	Pregnenetriol disulfate	5	5.574	0.233	0.958	6.04E-19
	N-acetylglucosaminylasparagine	5	4.332	0.363	0.282	9.93E-08
	Beta-hydroxyisovalerate	4	2.553	0.466	0.471	0.155
	1-palmitoyl-2-oleoyl-GPE (16:0/18:1)	6	4.715	0.452	0.997	1.81E-05
	N6,n6,n6-trimethyllysine	3	0.305	0.858	0.679	0.121
	X-11470	6	3.448	0.631	0.903	0.0005
	X-24588	6	8.801	0.117	0.424	0.026
	X-25371	4	0.925	0.819	0.506	0.003
	Deoxycholic acid glucuronide	3	0.519	0.771	0.795	1.37E-67
	Aspartate to N-acetylglucosamine to N-acetylgalactosamine ratio	3	1.481	0.477	0.438	0.078
	Retinol to oleoyl-linoleoyl-glycerol (18:1 to 18:2) [2] ratio	6	4.529	0.476	0.922	0.088
	N-acetylneuraminate to N-acetylglucosamine to N-acetylgalactosamine ratio	3	2.575	0.276	0.365	0.002
ALL	Kynurenine	4	2.843	0.416	0.374	0.063
	Behenoyl dihydrosphingomyelin (d18:0/22:0)	4	0.965	0.810	0.435	0.261
	1-palmitoyl-2-docosahexaenoyl-GPE (16:0/22:6)	5	1.692	0.792	0.521	8.24E-13
	1-stearoyl-2-docosahexaenoyl-GPE (18:0/22:6)	4	3.282	0.350	0.825	8.81E-05
	1-stearoyl-2-linoleoyl-GPI (18:0/18:2)	4	2.912	0.405	0.595	0.003
	1-stearoyl-2-oleoyl-GPI (18:0/18:1)	5	3.076	0.545	0.328	0.017
	1-palmitoyl-2-oleoyl-GPI (16:0/18:1)	4	2.675	0.444	0.548	0.077
	Cerotoylcarnitine (C26)	7	2.854	0.827	0.515	0.002
	N-acetylglucosaminylasparagine	5	1.391	0.846	0.727	9.89E-08
	Beta-hydroxyisovalerate	4	1.055	0.788	0.505	0.155
	X-24588	6	6.677	0.246	0.760	0.026
	Retinol to oleoyl-linoleoyl-glycerol (18:1 to 18:2) [2] ratio	6	2.918	0.713	0.650	0.088
CLL	Beta-hydroxyisovaleroylcarnitine	3	1.047	0.592	0.925	0.007
	5alpha-pregnan-3beta,20alpha-diol disulfate	3	0.225	0.893	0.720	0.009
	Pregnenediol disulfate (C21H34O8S2)	6	1.201	0.945	0.584	5.38E-16
	Palmitoyl sphingomyelin (d18:1/16:0)	4	0.339	0.953	0.901	0.455
	Dihomo-linoleoylcarnitine (C20:2)	4	0.783	0.853	0.985	0.0003
	Eicosenoylcarnitine (C20:1)	3	0.480	0.786	0.665	0.278
	Gamma-glutamylcitrulline	4	2.926	0.403	0.541	0.420
	N6,n6,n6-trimethyllysine	3	1.270	0.530	0.473	0.121
	X-11470	6	4.220	0.518	0.401	0.0005
	Decadienedioic acid (C10:2-DC)	3	1.074	0.585	0.495	0.101
	Carnitine to ergothioneine ratio	3	0.975	0.614	0.574	0.036

NSNP refers to the number of SNPs used for analysis; the Q statistic is a statistical measure of heterogeneity, with a higher value indicating a more significant difference between study results.

**Table 3:** Mendelian randomization analyses of the causal effects between immune cells and Lymphoid leukaemia.

Outcome	Exposure	N snp	Q statistic	P heterogeneity	P intercept	Steiger pval
Lymphoid leukaemia	CD62L- plasmacytoid Dendritic Cell %Dendritic Cell	3	0.560	0.756	0.591	0.003
	Secreting CD4 regulatory T cell %CD4 regulatory T cell	5	2.831	0.587	0.333	9.98E-05
	Activated & resting CD4 regulatory T cell %CD4 regulatory T cell	5	2.864	0.581	0.328	8.77E-05
	Monocytic Myeloid-Derived Suppressor Cells Absolute Count	5	4.375	0.358	0.201	1.44E-05
	Basophil %CD33dim HLA DR- CD66b-	3	1.169	0.557	0.548	3.02E-05
	HLA DR+ T cell Absolute Count	3	0.401	0.818	0.714	6.52E-05
	HLA DR+ T cell%T cell	4	1.703	0.636	0.646	4.93E-07
	HLA DR+ CD8+ T cell Absolute Count	3	0.412	0.814	0.691	0.0002
	HLA DR+ CD8+ T cell %T cell	3	0.474	0.789	0.711	8.15E-06
	CD45RA- CD28- CD8+ T cell %T cell	35	35.780	0.385	0.261	6.31E-06
	IgD on IgD+ CD24- B cell	5	3.732	0.444	0.786	3.23E-13
	IgD on IgD+ CD38dim B cell	5	3.678	0.451	0.831	1.35E-10
	CD16-CD56 on Natural Killer	7	8.651	0.194	0.768	1.47E-31
	CD33 on CD14+ monocyte	6	3.364	0.644	0.472	4.58E-72
	CD33 on CD33+ HLA DR+ CD14dim	4	1.181	0.757	0.673	8.49E-69
	CD33 on CD33dim HLA DR+ CD11b+	7	3.493	0.745	0.413	3.90E-73
	CD33 on CD33dim HLA DR+ CD11b-	6	1.938	0.858	0.484	3.11E-68
	CD33 on Granulocytic Myeloid-Derived Suppressor Cells	3	1.078	0.583	0.600	7.74E-12
	CD33 on CD66b++ myeloid cell	5	1.304	0.861	0.590	4.85E-30
	CD33 on Monocytic Myeloid-Derived Suppressor Cells	3	0.481	0.786	0.761	1.08E-50
	CD33 on CD33dim HLA DR-	6	4.425	0.490	0.963	2.83E-47
	CD33 on basophil	7	6.477	0.372	0.762	6.46E-51
	CD33 on Immature Myeloid-Derived Suppressor Cells	6	6.067	0.300	0.658	2.72E-46
	CD33 on CD33+ HLA DR+	4	1.193	0.755	0.664	5.41E-71
	CD33 on CD33+ HLA DR+ CD14-	4	1.181	0.758	0.661	4.70E-72
	FSC-A on plasmacytoid Dendritic Cell	4	2.789	0.425	0.428	0.160
	HLA DR on monocyte	5	3.342	0.502	0.545	2.51E-12
	SSC-A on plasmacytoid Dendritic Cell	4	2.775	0.428	0.369	0.116
	HLA DR on B cell	9	7.519	0.482	0.748	9.72E-15
	CD45RA+ CD28- CD8+ T cell Absolute Count	16	21.243	0.129	0.811	0.009
ALL	CD62L- plasmacytoid Dendritic Cell %Dendritic Cell	3	0.994	0.608	0.962	0.003
	CD28+ CD45RA+ CD8dim T cell Absolute Count	8	6.900	0.439	0.542	0.015
	CD45RA+ CD28- CD8+ T cell %T cell	60	46.960	0.871	0.163	8.93E-10
	CD38 on IgD+ CD38+ B cell	3	2.463	0.292	0.361	0.149
	CD38 on IgD+ CD38dim B cell	4	1.398	0.706	0.777	0.009
	CD38 on IgD- CD38dim B cell	3	0.367	0.833	0.763	0.032
	CD38 on naive-mature B cell	3	0.431	0.806	0.800	0.076
	CD38 on transitional B cell	5	2.556	0.635	0.424	0.007
	CD3 on Effector Memory CD8+ T cell	3	1.014	0.602	0.876	0.005
	FSC-A on Natural Killer	3	2.115	0.347	0.448	0.070
	HLA DR on CD14- CD16+ monocyte	5	2.173	0.704	0.528	1.36E-07
	CD80 on granulocyte	3	1.130	0.568	0.519	0.096
CLL	Switched memory B cell %lymphocyte	3	2.369	0.306	0.395	0.314
	HLA DR+ T cell Absolute Count	3	1.107	0.575	0.486	6.54E-05
	HLA DR+ T cell%T cell	4	3.371	0.338	0.505	4.98E-07
	HLA DR+ CD8+ T cell Absolute Count	3	1.287	0.525	0.462	0.0002
	HLA DR+ CD8+ T cell %T cell	3	0.349	0.840	0.674	8.14E-06
	CD45RA- CD28- CD8+ T cell %CD8+ T cell	10	11.319	0.254	0.561	0.009
	BAFF-R on IgD+ CD38- naive B cell	7	2.600	0.857	0.507	2.27E-63
	CD25 on IgD+ CD38+ B cell	3	1.765	0.414	0.613	0.087
	IgD on IgD+ CD38+ B cell	4	0.696	0.874	0.581	9.25E-17
	IgD on transitional B cell	4	3.072	0.381	0.672	3.56E-08
	CD16-CD56 on Natural Killer	7	9.097	0.168	0.387	1.48E-31
	FSC-A on plasmacytoid Dendritic Cell	4	2.359	0.501	0.270	0.160
	SSC-A on plasmacytoid Dendritic Cell	4	0.784	0.853	0.713	0.116

NSNP refers to the number of SNPs used for analysis; the Q statistic is a statistical measure of heterogeneity, with a higher value indicating a more significant difference between study results.

### 3.6 Directionality test

We performed Steiger tests to explore potential reverse causal relationships between gut microbiota, metabolites, immune cells, and lymphoid leukemia, including ALL and CLL. *P*<0.05 suggest that the Steiger test results do not support reverse causal effects among these variables (**Tables 1**-**3**).

### 3.7 Mediating role of serum metabolites in immune cells and lymphoid leukaemia

We used the IVW method to identify immune cells and serum metabolites with significant causal associations in lymphoid leukaemia, ALL, and CLL (**Supplementary Table 5**). We further conducted a two-step MR analysis on the relevant serum metabolites and immune cells. We found that there are different mediating effects in the aforementioned lymphoid leukaemia, ALL, and CLL.

**Figure 5:**
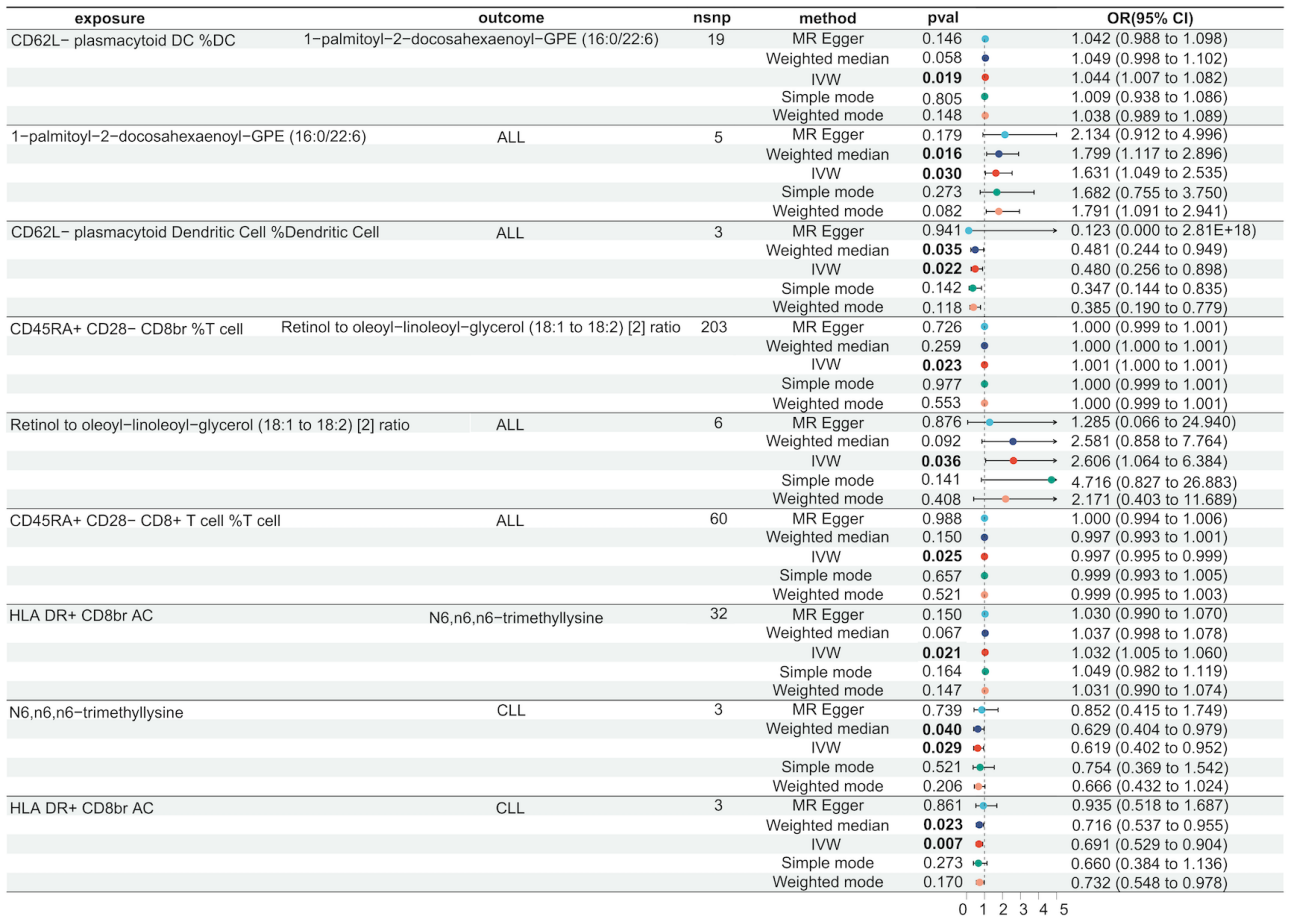
Serum metabolites as intermediates in causal effects of ALL and CLL on Immune Cells.

**Figure 6:**
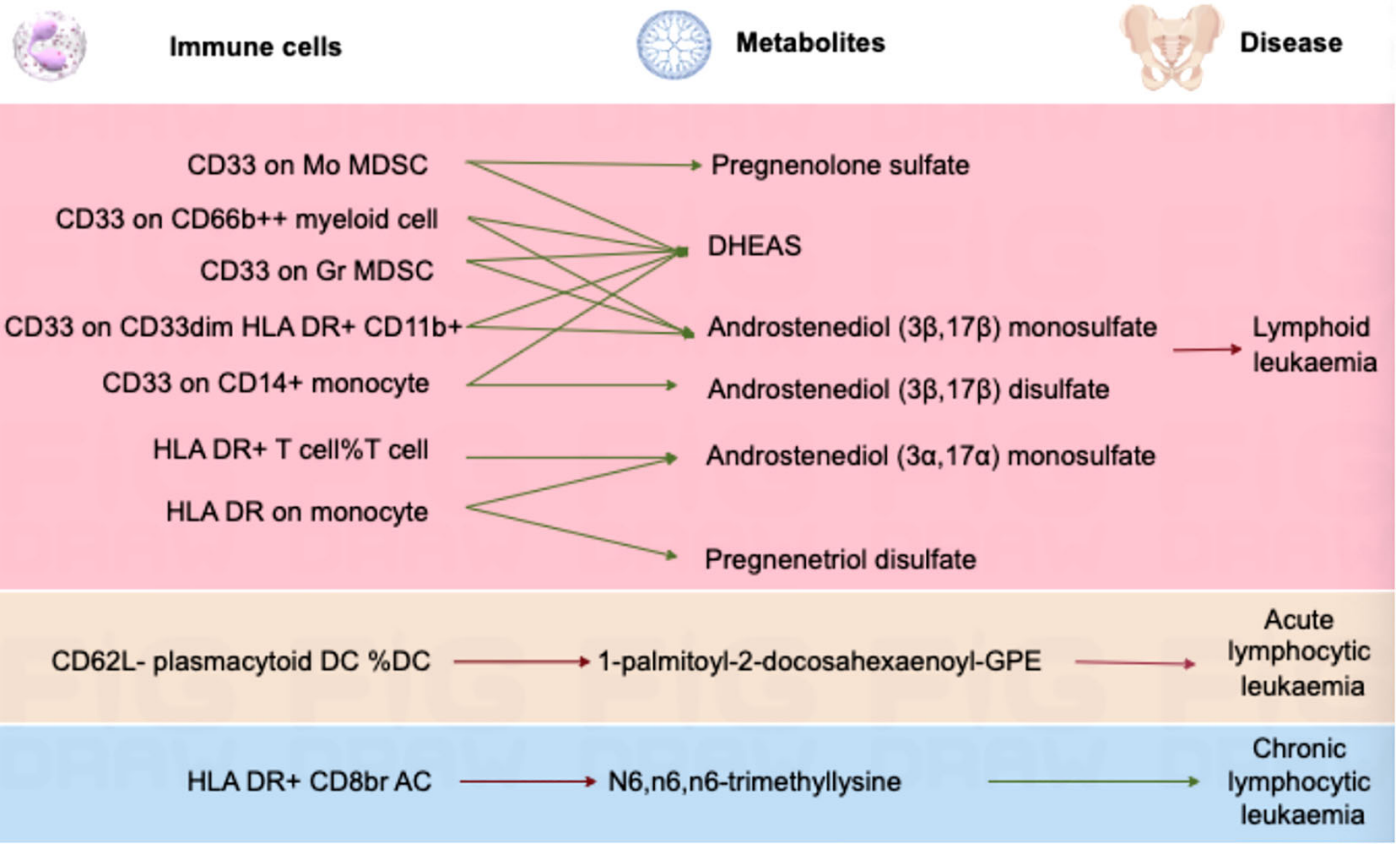
Mendelian randomization analysis shows the causal effects of serum metabolites on immune cells and lymphoid leukaemia,ALL,CLL.This figure illustrates the mediation model of “immune cells—serum metabolites—lymphoid leukaemia” in a two-step Mendelian randomization. Red and green characters represent positive (β>0) and negative (β<0) associations, respectively.

In lymphoid leukaemia, CD62L- plasmacytoid DC %DC mediated the causal relationship between 1-palmitoyl-GPE (16:0) (Mediated proportion = -8.35%[-3.65%,-13.1%]) and lymphoid leukaemia. HLA DR+ T cell %T cell mediated the effects of 1-palmitoyl-GPE (16:0) (Mediated proportion = -5.12%[-0.532%,-9.7%]), Androstenediol (3alpha, 17alpha) monosulfate (Mediated proportion = 2.75%[5.34%, 0.156%]), 1-stearoyl-2-linoleoyl-GPI (18:0/18:2) (Mediated proportion = 4.05%[8.03%,0.061%]), and Aspartate to N-acetylglucosamine to N-acetylgalactosamine ratio (Mediated proportion = -7.52%[-1.46%, -13.6%]). HLA DR+ CD8br AC mediated the causal relationship between N6,n6,n6-trimethyllysine (Mediated proportion = 5.39%[9.96%,0.807%]), Aspartate to N-acetylglucosamine to N-acetylgalactosamine ratio (Mediated proportion = -5.69% [-0.737%, -10.7%]), and lymphoid leukaemia. HLA DR+ CD8br %T cell mediated the causal relationship between X-24588 (Mediated proportion = 6.71%[12.2%,1.24%]), X-25371 (Mediated proportion = -4.36% [-0.429%, -8.3%]), and lymphoid leukaemia. CD45RA- CD28- CD8br %T cell mediated the causal relationship between 1-stearoyl-2-linoleoyl-GPI (18:0/18:2) and lymphoid leukaemia (Mediated proportion = -8.31%[-15.7%,-0.872%]). CD45RA+ CD28- CD8br AC mediated the causal relationship between Deoxycholic acid glucuronide and lymphoid leukaemia (Mediated proportion = -0.271%[-0.493%, -0.049%]). IgD on IgD+ CD24- mediated the causal relationship between 1-palmitoyl-GPE (16:0) and lymphoid leukaemia (Mediated proportion = -13% [-1.24%,-24.7%]). CD33 on CD14+ monocyte mediated the causal relationship between DHEAS (Mediated proportion = -9.72%[-17.8%,-1.65%]), Androstenediol (3beta,17beta) disulfate (Mediated proportion = -14.6% [-26.9%,-2.27%]), and lymphoid leukaemia. CD33 on CD33dim HLA DR+ CD11b+ mediated the causal relationship between DHEAS (Mediated proportion = -8.22%[-16.4%,-0.035%]), Androstenediol (3beta,17beta) monosulfate (Mediated proportion = -11%[-21.9%,-0.219%]), Aspartate to N-acetylglucosamine to N-acetylgalactosamine ratio (Mediated proportion = -13.5%[-25.4%,-1.68%]), and lymphoid leukaemia. CD33 on Gr MDSC mediated the causal relationship between DHEAS (Mediated proportion = -9.56%[-16%,-3.16%]), Androstenediol (3beta,17beta) monosulfate (Mediated proportion = -12.3%[-20.7%, -3.83%]), and lymphoid leukaemia. CD33 on CD66b++ myeloid cell mediated the causal relationship between DHEAS (Mediated proportion = -7.86%[-15.1%,-0.631%]), Androstenediol (3beta,17beta) monosulfate (Mediated proportion = -11.8%[-21.3%, -2.25%]), Aspartate to N-acetylglucosamine to N-acetylgalactosamine ratio (Mediated proportion = -12%[-22.5%,-1.55%]), and lymphoid leukaemia. CD33 on Mo MDSC mediated the causal relationship between DHEAS (Mediated proportion = -9.67%[-19.4%,0.025%]), Pregnenolone sulfate (Mediated proportion = -9.2%[-17.4%,-0.965%]), and lymphoid leukaemia. CD33 on CD33dim HLA DR- mediated the causal relationship between Retinol to oleoyl-linoleoyl-glycerol (18:1 to 18:2) [2] ratio and lymphoid leukaemia (Mediated proportion = -13%[-23.5%, -2.55%]). CD33 on Im MDSC mediated the causal relationship between Retinol to oleoyl-linoleoyl-glycerol (18:1 to 18:2) [2] ratio and lymphoid leukaemia (Mediated proportion = -13.1% [-23.1%,-3.09%]). HLA DR on monocyte mediated the causal relationship between Androstenediol (3alpha, 17alpha) monosulfate (Mediated proportion = 5.43%[10.7%, 0.176%]), Pregnenetriol disulfate (Mediated proportion = 8.42%[16.7%,0.192%]), and lymphoid leukaemia. HLA DR on B cell mediated the causal relationship between Dihomo-linoleoylcarnitine (C20:2) (Mediated proportion = 9.12% [1.87%, 16.4%]), 1-palmitoyl-2-oleoyl-GPE (16:0/18:1) (Mediated proportion = -6.38%[-12.3%,-0.405%]), and lymphoid leukaemia.We further discovered the presence of various steroid hormone metabolites (such as DHEAS, progestogen derivatives, and androstenediol-related compounds) as potential intermediary metabolites between lymphatic leukaemia and immune cells. In ALL, CD62L- plasmacytoid DC %DC mediated the causal relationship between 1-palmitoyl-2-docosahexaenoyl-GPE (16:0/22:6) and ALL (Mediated proportion = -2.84%[-0.456%,-5.23%]). In CLL, HLA DR+ CD8br AC mediated the causal relationship between N6,n6,n6-trimethyllysine and CLL (Mediated proportion = 4.07%[7.53%,0.615%]) (**Figures 5**, **6**, **Table 4** and **Supplementary Figure 6**).

**Table 4:** Two-step Mendelian randomization analyses of the causal effects between immune cells, serum metabolites and Lymphoid leukaemia.

Immune Cell	Metabolite	Outcome	Mediated Effect	Mediated Proportion	Pval
CD62L- plasmacytoid DC %DC	1-palmitoyl-GPE (16:0)	Lymphoid leukaemia	0.020 (0.009, 0.031)	-8.35% (-3.65%, -13.1%)	0.0005
	X-25371		-0.016 (-0.027, -0.004)	6.47% (11.4%, 1.49%)	0.011
HLA DR+ T cell%T cell	1-palmitoyl-GPE (16:0)		0.008 (0.0009, 0.016)	-5.12% (-0.532%, -9.7%)	0.029
	Androstenediol (3alpha, 17alpha) monosulfate		-0.004 (-0.009, -0.0003)	2.75% (5.34%, 0.156%)	0.038
	1-stearoyl-2-linoleoyl-GPI (18:0/18:2)		-0.007 (-0.013, -9.85e-05)	4.05% (8.03%, 0.061%)	0.047
	Aspartate to N-acetylglucosamine to N-acetylgalactosamine ratio		0.012 (0.002, 0.022)	-7.52% (-1.46%, -13.6%)	0.015
HLA DR+ CD8br AC	N6,n6,n6-trimethyllysine		-0.012 (-0.022, -0.002)	5.39% (9.96%, 0.807%)	0.021
	Aspartate to N-acetylglucosamine to N-acetylgalactosamine ratio		0.013 (0.002, 0.024)	-5.69% (-0.737%, -10.7%)	0.024
HLA DR+ CD8br %T cell	X-24588		-0.014 (-0.025, -0.003)	6.71% (12.2%, 1.24%)	0.016
	X-25371		0.009 (0.0009, 0.017)	-4.36% (-0.429%, -8.3%)	0.030
CD45RA- CD28- CD8br %T cell	1-stearoyl-2-linoleoyl-GPI (18:0/18:2)		-0.0002 (-0.0003,-1.74e-05)	-8.31% (-15.7%, -0.872%)	0.029
CD45RA+ CD28- CD8br AC	Deoxycholic acid glucuronide		-2.71e-06 (-4.93e-06, -4.85e-07)	-0.271% (-0.493%, -0.049%)	0.017
IgD on IgD+ CD24-	1-palmitoyl-GPE (16:0)		0.015 (0.001, 0.028)	-13% (-1.24%, -24.7%)	0.030
CD33 on CD14+ monocyte	DHEAS		-0.007 (-0.013, -0.001)	-9.72% (-17.8%, -1.65%)	0.018
	Androstenediol (3beta,17beta) disulfate		-0.011 (-0.020, -0.002)	-14.6% (-26.9%, -2.27%)	0.020
CD33 on CD33dim HLA DR+ CD11b+	DHEAS		-0.006 (-0.012, -2.47e-05)	-8.22% (-16.4%, -0.035%)	0.049
	Androstenediol (3beta,17beta) monosulfate		-0.008 (-0.016, -0.0002)	-11% (-21.9%, -0.219%)	0.046
	Aspartate to N-acetylglucosamine to N-acetylgalactosamine ratio		-0.010 (-0.018, -0.001)	-13.5% (-25.4%, -1.68%)	0.025
CD33 on Gr MDSC	DHEAS		-0.010 (-0.016, -0.003)	-9.56% (-16%, -3.16%)	0.003
	Androstenediol (3beta,17beta) monosulfate		-0.013 (-0.021, -0.004)	-12.3% (-20.7%, -3.83%)	0.004
CD33 on CD66b++ myeloid cell	DHEAS		-0.007 (-0.013, -0.0005)	-7.86% (-15.1%, -0.631%)	0.033
	Androstenediol (3beta,17beta) monosulfate		-0.010 (-0.018, -0.002)	-11.8% (-21.3%, -2.25%)	0.015
	Aspartate to N-acetylglucosamine to N-acetylgalactosamine ratio		-0.010 (-0.019, -0.001)	-12% (-22.5%, -1.55%)	0.024
CD33 on Mo MDSC	DHEAS		-0.007 (-0.014, 1.87e-05)	-9.67% (-19.4%, 0.025%)	0.050
	Pregnenolone sulfate		-0.007 (-0.013, -0.0007)	-9.2% (-17.4%, -0.965%)	0.029
CD33 on CD33dim HLA DR-	Retinol to oleoyl-linoleoyl-glycerol (18:1 to 18:2) [2] ratio		-0.010 (-0.018, -0.002)	-13% (-23.5%, -2.55%)	0.015
CD33 on Im MDSC	Retinol to oleoyl-linoleoyl-glycerol (18:1 to 18:2) [2] ratio		-0.011 (-0.019, -0.0025)	-13.1% (-23.1%, -3.09%)	0.010
HLA DR on monocyte	Androstenediol (3alpha, 17alpha) monosulfate		-0.005 (-0.011, -0.0002)	5.43% (10.7%, 0.176%)	0.043
	Pregnenetriol disulfate		-0.008 (-0.016, -0.0002)	8.42% (16.7%, 0.192%)	0.045
HLA DR on B cell	Dihomo-linoleoylcarnitine (C20:2)		0.009 (0.002, 0.016)	9.12% (1.87%, 16.4%)	0.014
	1-palmitoyl-2-oleoyl-GPE (16:0/18:1)		-0.006 (-0.012, -0.0004)	-6.38% (-12.3%, -0.405%)	0.036
CD62L- plasmacytoid DC %DC	1-palmitoyl-2-docosahexaenoyl-GPE (16:0/22:6)	ALL	0.021 (0.003, 0.038)	-2.84% (-0.456%, -5.23%)	0.020
HLA DR+ CD8br AC	N6,n6,n6-trimethyllysine	CLL	-0.015 (-0.028,-0.002)	4.07% (7.53%, 0.615%)	0.021

## 4 Discussion

In this study, we utilized large-scale GWAS summary data to perform comprehensive bidirectional two-sample MR and mediation analyses. These analyses investigated the causal relationships between gut microbiota, immune cells, serum metabolites, and lymphoid leukaemia, such as ALL and CLL.Two-step MR analysis is a method that enhances causal inference by using two independent genetic instrumental variables to verify the causal relationships between gut microbiota, immune cells, serum metabolites, and diseases. This can enhance the reliability of causal inference, improve the robustness of the analysis, reduce the risk of bias, and allow for the detection of multiple causal relationships. This method enables us to more precisely identify and validate the role of gut microbiota, immune cells, and serum metabolites in the development of diseases, thereby providing stronger evidence for support.Additionally, we aimed to delineate the mediating role of serum metabolites in the interaction between immune cells and lymphoid leukaemia.

Analysis of the causal relationship between gut microbiota and lymphocytic leukemia (ALL and CLL) revealed the presence of *Firmicutes* and *Bacteroidetes phyla* in all three diseases. Studies indicate that leukemia patients exhibit oral microbiota dysbiosis, with changes in the abundance of *Firmicutes* and *Bacilli* associated with leukemia status, specifically showing a significant 0.1% increase in *Firmicutes*. Oral microbial dysbiosis is also observed in ALL patients (33, 34). The gut microbiota of children with ALL shows greater inter-individual variability and is enriched with bacteria belonging to the *Bacteroidetes phylum* and *Bacteroides genus (* 35). In children receiving treatment for newly diagnosed ALL, the relative abundance of certain bacterial groups (e.g., *Bacteroidetes*) significantly decreased post-chemotherapy, while others (e.g., *Clostridiaceae* and *Streptococcaceae*) increased. A baseline gut microbiota characterized by *Proteobacteria* predicts febrile neutropenia (36).

Analysis of the causal relationship between serum metabolites and lymphocytic leukemia (ALL and CLL) revealed common metabolic pathways, including glycerophospholipid metabolism (e.g., 1-palmitoyl-2-docosahexaenoyl-GPE, 1-stearoyl-2-docosahexaenoyl-GPE), suggesting a central role for lipid signaling and cell membrane composition in lymphocytic leukemia. Sphingolipid metabolism (e.g., palmitoyl sphingomyelin) also plays a critical role in cell signaling and cell fate. Additionally, steroid hormone metabolism, involving pregnane metabolites and DHEAS, is crucial for regulating immune responses and hormone levels, potentially affecting leukemia cell survival. Energy and amino acid metabolism are also significant, with carnitine derivatives (e.g.,β-hydroxyisovaleroylcarnitine and eicosenoylcarnitine) involved in fatty acid transport and oxidation, influencing energy balance and cell survival.Increasing evidence suggests that adipocytes play an active role in the cancer microenvironment, and studying lipid and metabolic profiles is increasingly recognized as valuable for understanding tumorigenesis and progression. In the presence of ALL cells, adipocytes release free fatty acids (FFAs), which ALL cells absorb and incorporate into triglycerides and phospholipids. Some of these lipids are stored in lipid droplets, which can be utilized under energy-deprived conditions. Adipocytes preferentially release monounsaturated FFAs, which can be attenuated by inhibiting the desaturase enzyme stearoyl-CoA desaturase-1 (SCD1) (37). Studies have identified new potential metabolic biomarkers for the TAL/LMO subgroup and provided a sub-classification of T-ALL cell lines within the same subgroup using LC/MS (38). We also discovered ALL-specific vitamin metabolism, such as the retinol to oleoyl-linoleoyl-glycerol ratio, which may reflect unique cell differentiation mechanisms in ALL. Research indicates that, compared to vitamin A-sufficient mice, regulatory T cells appear more frequently in the CD4^+^ splenocytes of vitamin A-deficient mice. Treatment of leukemia cells with vitamin A (all-trans retinoic acid, ATRA) increases apoptosis, decreases S-phase cells, and increases G0/G1 phase cells. ATRA signals through the retinoid X receptor, reducing the viability of BCR-ABL leukemia cells.In CLL, unique small molecule metabolites such as N-acetyltaurine and gamma-glutamylcitrulline have been identified, which may be more significantly associated with oxidative stress and amino acid regulation. Studies have found that TP53 mutations in CLL lead to changes in amino acids, inhibiting leukemia cell apoptosis (39). The proliferation of primary CLL cells depends on the availability of extracellular arginine, with cationic amino acid transporter 1 (CAT-1) as the only arginine input protein expressed in CLL cells. Lentivirus-mediated downregulation of the CAT-1 transporter protein in HG3 CLL cells significantly reduces arginine uptake, eliminates cell proliferation, and impairs cell viability (40).

Through analyzing the causal relationship between immune cells and lymphoid leukemia, including ALL and CLL, commonalities with the following types of immune cells were identified. Among them, the CD8^+^ T cell subpopulation might be related to its cytotoxic function and potential to counteract tumor cells (41). Dendritic cells play a central role in initiating and regulating immune responses and might be crucial in combating viral infections and lymphoid leukemia tumor cells. In lymphoid leukemia, regulatory T cells were found to directly interact with other immune cells by secreting immunosuppressive factors, helping to maintain immune tolerance and prevent autoimmune reactions (42). Myeloid-derived suppressor cells (MDSCs) might promote immune evasion by inhibiting the function of T cells and NK cells, aiding tumor cells in lymphoid leukemia to escape immune surveillance (43). Various T cells and related subpopulations, monocytes and myeloid cells, B cells, natural killer cells, and basophils were found to be associated with lymphoid leukemia. The expression of CD33 and HLA-DR reflects the activation state and function of cells, playing a significant role in immune regulation (22). In ALL, causal relationships with various B cells were discovered, with CD38 being a marker of maturity and activation state. Different levels of CD38 expression help distinguish the developmental stages and functional states of B cells (44). Naive B cells expressing BAFF-R are specifically mentioned in CLL and might be related to abnormalities in the survival and maturation processes of B cells in CLL (45). CD127-expressing CD4^+^ T cells are mentioned in CLL, potentially associated with long-term immune surveillance and chronic inflammatory states (46).

Our mediation analysis provided genetic evidence indicating that different serum metabolites mediate the effects of immune cells on lymphoid leukemia.A mediation Mendelian analysis conducted in lymphoid leukemia revealed that DHEAS influences the expression of CD33 on various immune cells, which may be related to the pathogenesis of lymphoid leukemia. These cells include CD14+ monocytes, CD33dim HLA DR+ CD11b+, Gr MDSC, CD66b++ myeloid cells, and Mo MDSC. DHEAS is a steroid hormone derived from the adrenal gland that can affect the differentiation, maturation, and release of various cytokines by immune cells, thereby influencing the overall immune response, the tumor microenvironment, and the immune evasion of tumor cells (47). CD33 is a marker expressed on various myeloid cells and is typically associated with the maturity and activation state of the cells (48). DHEAS may indirectly affect the transcription of the CD33 gene by regulating the activity of related transcription factors. DHEAS can influence the inflammatory response by modulating the production of inflammatory factors such as TNF-α and IL-6, thereby impacting the progression of leukemia (49). Additionally, DHEAS may regulate the expression of CD33 and other immune regulatory factors through epigenetic mechanisms. By altering the state of DNA methylation or histone modifications, DHEAS may indirectly regulate the expression of multiple immune-related genes (50).

In ALL, we found that 1-palmitoyl-2-docosahexaenoyl-GPE (16:0/22:6) mediates the relationship between CD62L- plasmacytoid DC %DC and ALL.1-palmitoyl-2-docosahexaenoyl-GPE is a glycerophospholipid that contains the long-chain polyunsaturated fatty acid docosahexaenoic acid (DHA). DHA is known to alter cell membrane fluidity and structure, which may affect the arrangement and function of cell surface receptors, including those involved in immune recognition and cell signaling (51). Study found that fatty acid metabolites can be present in immune cells and participate in signal transduction in leukemia cells (52). Plasmacytoid DCs are important producers of interferons, especially in antiviral immune responses. Changes in lipid molecules can regulate the activation state of transcription factors such as NF-κB. DHA and other polyunsaturated fatty acids have also been shown to regulate intracellular signaling pathways such as PI3K/AKT and MAPK, thereby influencing the production of interferons and other cytokines (53). This, in turn, may alter the immune system’s ability to monitor and eliminate ALL.

In CLL, it was found that N6,n6,n6-trimethyllysine mediates the relationship between HLA DR+ CD8br AC and CLL. N6,n6,n6-trimethyllysine is a methylation modification occurring on lysine residues. This modification can affect the three-dimensional structure, stability, interactions with other proteins, or cellular localization of proteins (54). In immune cells, such modifications may influence the function of key signaling molecules, such as those involved in T cell receptor (TCR) complex signaling, thereby affecting the activation, proliferation, and cytotoxic function of CD8^+^ T cells.Methylated lysine can alter the function of proteins such as histones, thereby affecting gene expression. In CD8^+^ T cells, this may lead to changes in cell phenotype, such as increased expression of HLA DR (55). The expression of HLA DR is typically associated with the activation state of cells; its expression in CD8^+^ T cells may indicate a highly activated state, which could be significant in combating CLL cells. HLA DR+ CD8+ T cells, due to their role in immune responses, particularly in presenting tumor antigens and activating cytotoxic responses, may have a crucial impact on the progression of CLL (56). N6,n6,n6-trimethyllysine, by influencing the phenotype and function of these cells, may enhance or alter their ability to recognize and eliminate CLL cells (57).

This study presents several innovative aspects. First, to our knowledge, it is the first to combine metabolomics and genomics to implement MR analysis, addressing the causal relationships between serum metabolites, immune cells, and lymphoid leukaemia. It examines the influence of serum metabolites on immune cells and lymphoid leukaemia, holding significant clinical research value and offering new avenues for developing targeted therapies.The study employs Mendelian Randomization to investigate the causal relationship between gut microbiota as an exposure factor and lymphoid leukaemia, aiming to elucidate changes in the gut microbiome of patients with ALL and CLL. This provides a foundation for further research on the role of the gut microbiome in lymphoid leukaemia. Moreover, this work examines the effects of serum metabolites on immune cells. The study employs multiple MR models and establishes strict quality control conditions, ensuring reliable and robust results.Finally, the study encompasses a vast array of exposure factors—1400 serum metabolites, 731 immune cells, and 418 gut microbes. The integration of bidirectional two-sample Mendelian analysis and mediation analysis adds complexity, presenting significant analytical challenges.

However, this study has several limitations. Although we utilized comprehensive serum metabolomics data from Canada, the range of metabolites studied was not exhaustive. Future GWAS should include a broader spectrum of metabolites to identify additional causal compounds. Furthermore, the availability of classification data for lymphoid leukaemia is limited, and GWAS summary data reflect lifetime genetic exposure, indicating a need for further clinical and animal studies to determine whether the causal inferences from MR analysis represent short-term effects.Additionally, our study population is predominantly of European descent, and genetic variations may differ significantly across global populations, leading to potential bias due to population stratification. This variability necessitates cautious interpretation of our results’ generalizability to other racial and ethnic groups. Future research should include more diverse populations to enhance the applicability of the findings.Lastly, Mendelian Randomization assumes a linear relationship between exposure and outcome, which may not capture the true complexity of these interactions, potentially involving nonlinear dynamics and interactions with other environmental and genetic factors (58). Therefore, it is crucial to thoroughly consider the potential nonlinear and interactive effects between exposure and outcome.

## 5 Conclusion

This study represents the first comprehensive evaluation of the causal relationships between gut microbiota, serum metabolites, immune cells, and lymphoid leukaemia, including ALL and CLL. Our findings underscore the importance of elucidating the underlying mechanisms linking immune cells and lymphoid leukaemia,including ALL and CLL. These results offer new insights into treating lymphoid leukaemia via the microbiota, as well as through immune cell-based therapies and metabolite-targeted interventions.

## Data Availability

The original contributions presented in the study are included in the article/**Supplementary Material**. Further inquiries can be directed to the corresponding authors.
