# Corrigendum: Causal pathways in lymphoid leukemia: the gut microbiota, immune cells, and serum metabolites

**DOI:** 10.3389/fimmu.2024.1498574

**Published:** 2024-11-07

**Authors:** Xin Zhuang, Qingning Yin, Rong Yang, Xiaoying Man, Ruochen Wang, Hui Geng, Yifen Shi

**Affiliations:** ^1^ Department of Hematology, The First Affiliated Hospital of Wenzhou Medical University, Wenzhou, China; ^2^ Department of Hematology, Qinghai Province Women and Children’s Hospital, Xining, Qinghai, China; ^3^ Department of Hematology, Affiliated Hospital of Qinghai University, Xining, Qinghai, China; ^4^ Department of Vice President, Qinghai Province Women and Children’s Hospital, Xining, Qinghai, China; ^5^ Zhejiang Provincial Clinical Research Center For Hematological Disorders, Wenzhou, China

**Keywords:** gut microbiota, immune cells, lymphocytic leukemia, Mendelian randomization analysis, serum metabolites

In the published article, there was an error in affiliation(s) for Hui Geng. Instead of “Department of Vice President, Qinghai Province Women and Children’s Hospital, Xining, Qinghai, China”, it should be “Department of Hematology, Affiliated Hospital of Qinghai University, Xining, Qinghai, China”.

In the published article, there was an error in affiliation(s) for Qingning Yin. Instead of “Department of Vice President, Qinghai Province Women and Children’s Hospital, Xining, Qinghai, China”, it should be “Department of Hematology, Qinghai Province Women and Children’s Hospital, Xining, Qinghai, China”.

The authors apologize for this error and state that this does not change the scientific conclusions of the article in any way. The original article has been updated.

