# Supplementary material for: Causal pathways in lymphoid leukemia: the gut microbiota, immune cells, and serum metabolites
Source: Front Immunol. 2024 Sep 16;15:1437869. doi: 10.3389/fimmu.2024.1437869 (PMC11439652; doi:10.3389/fimmu.2024.1437869)
Supplement: Supplementary file 1 [file DataSheet1.zip › Supplementary Figure 4.pdf]

A

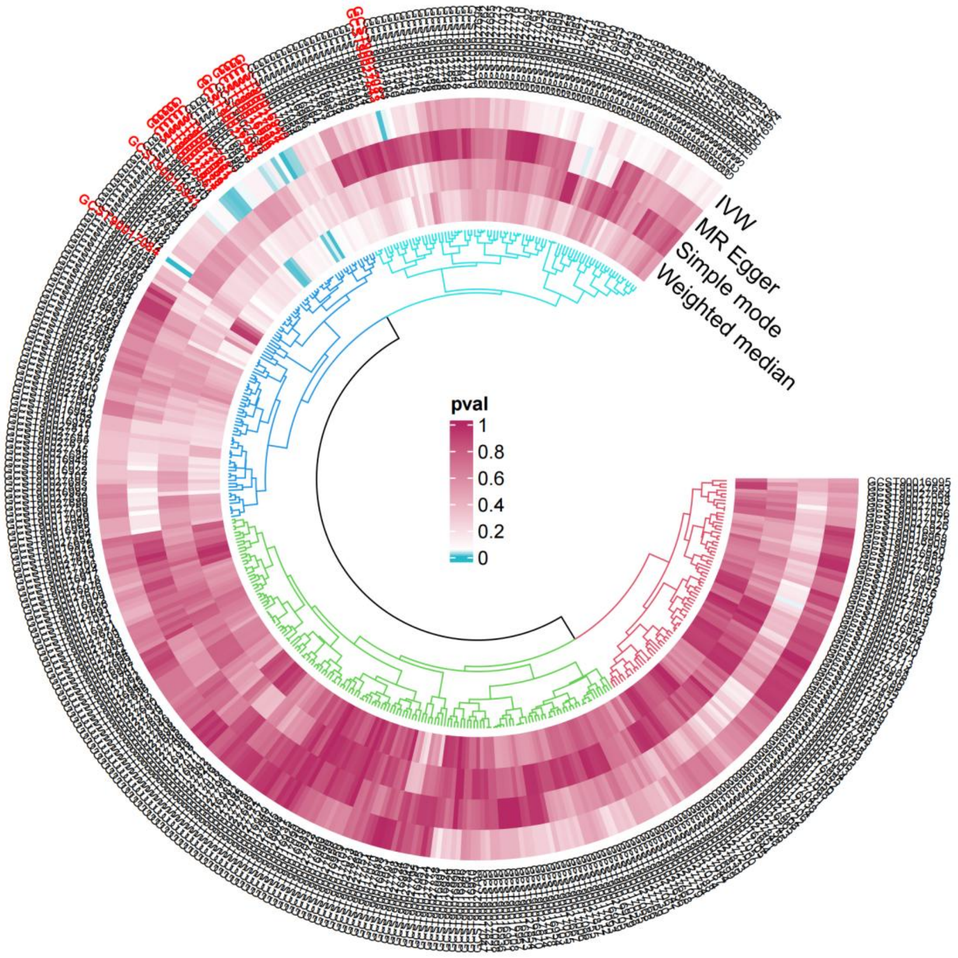
IVW
MR Egger
Simple mode
Weighted median
pval
1
0.8
0.6
0.4
0.2
0
GCST90016995

B

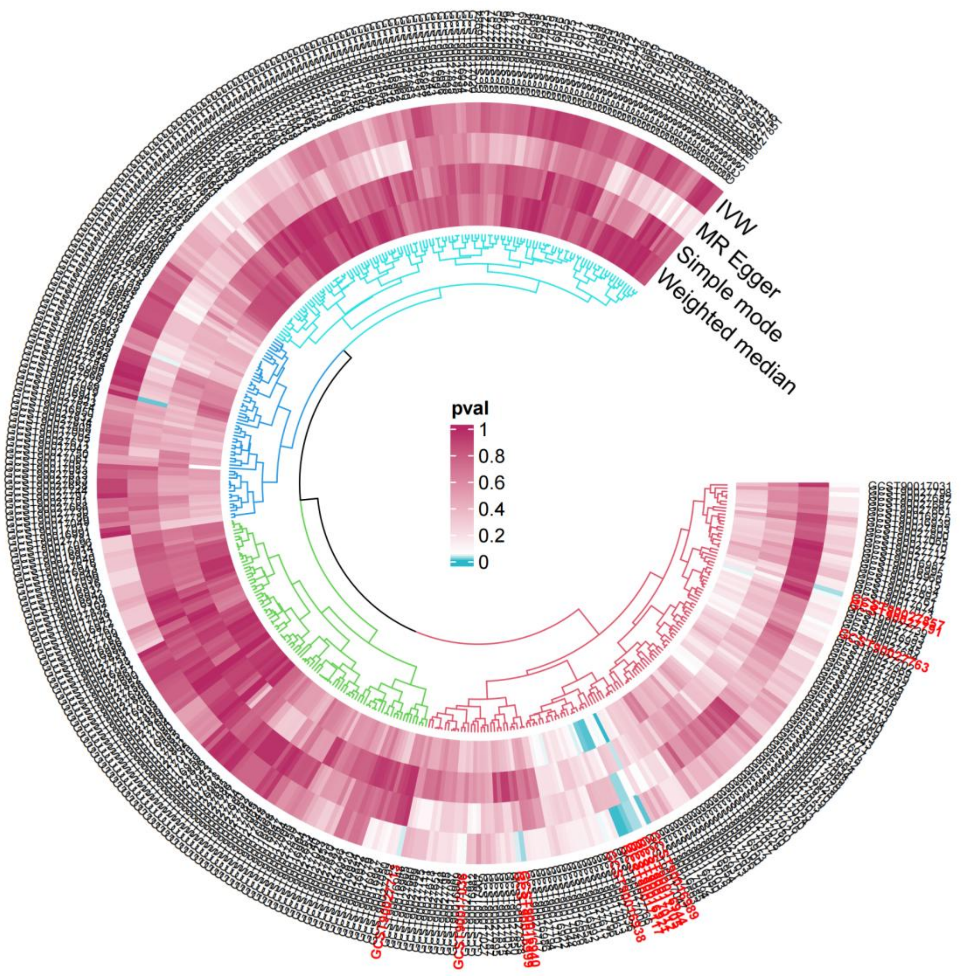

C

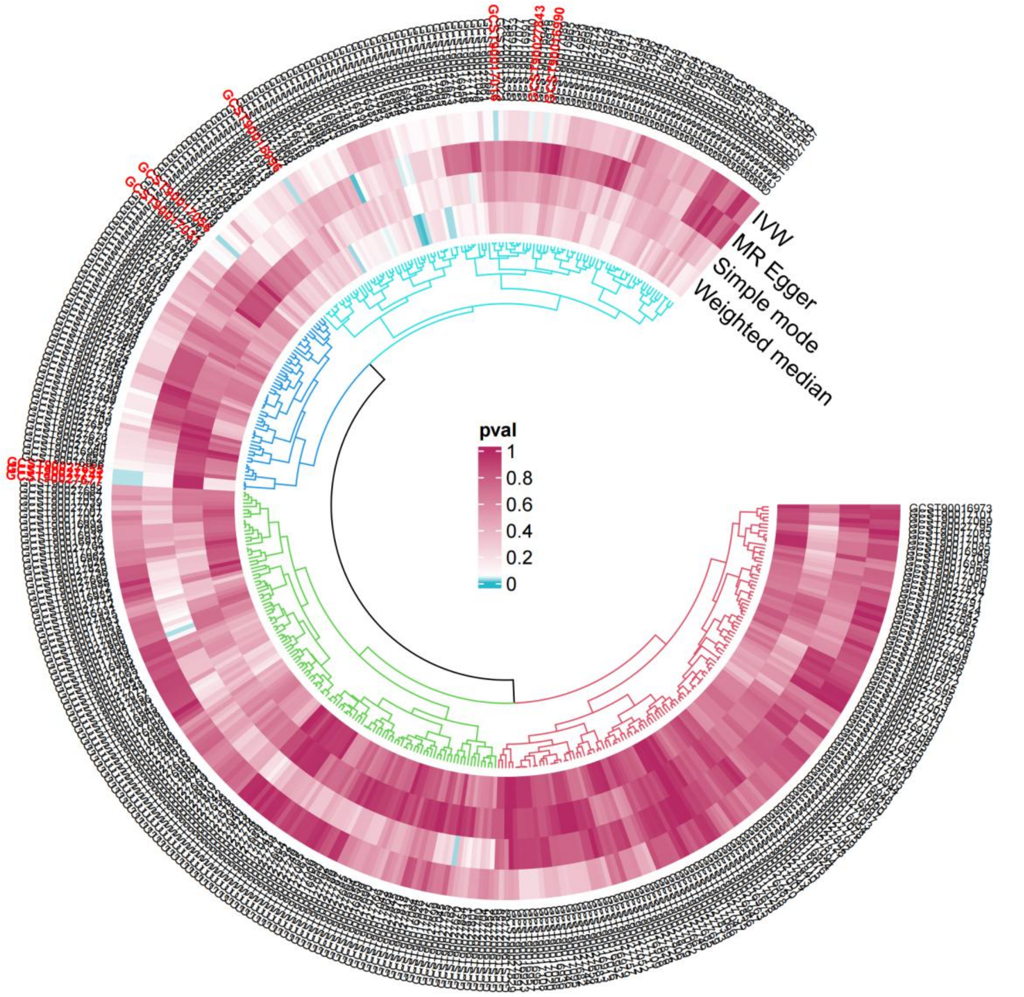

Supplement4 S1|Circular heat map of suggestive genetic correlation between Gut microbiota and(A)Lymphoid leukaemia (B)ALL (C)CLL.

A

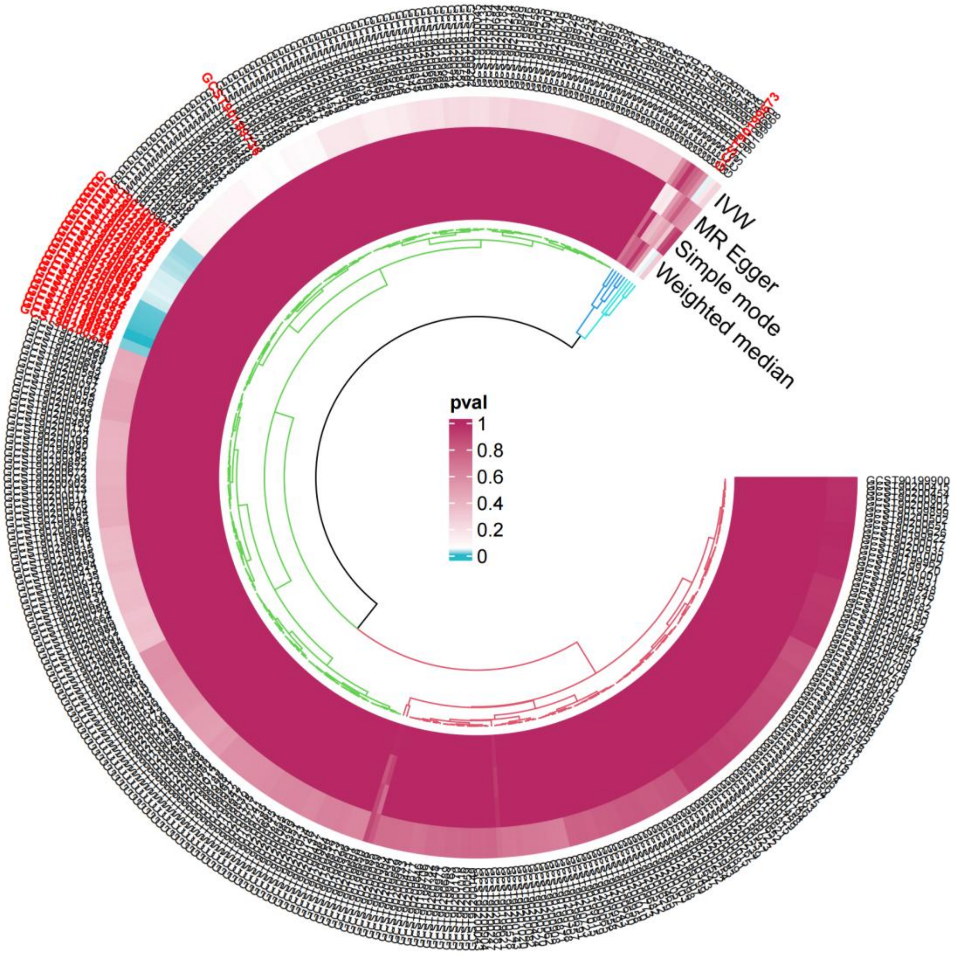

B

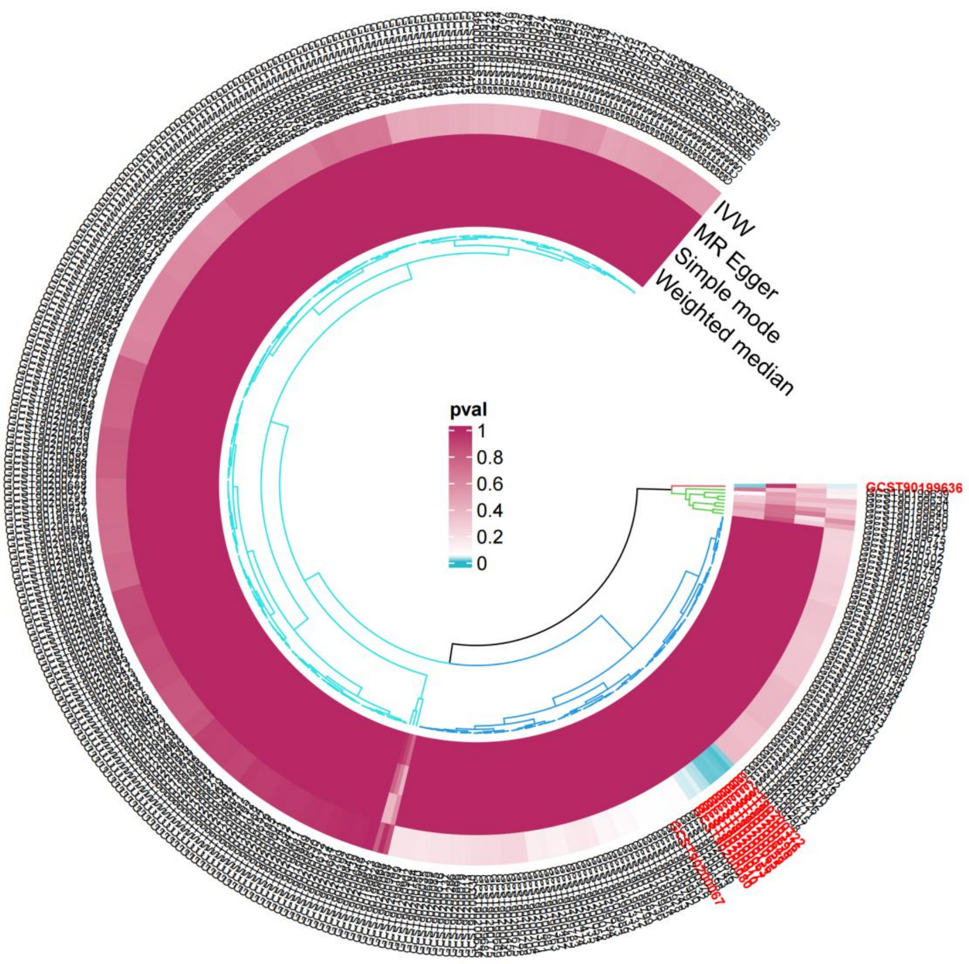
IVW
MR Egger
Simple mode
Weighted median
pval
1
0.8
0.6
0.4
0.2
0
GCST90199636

C

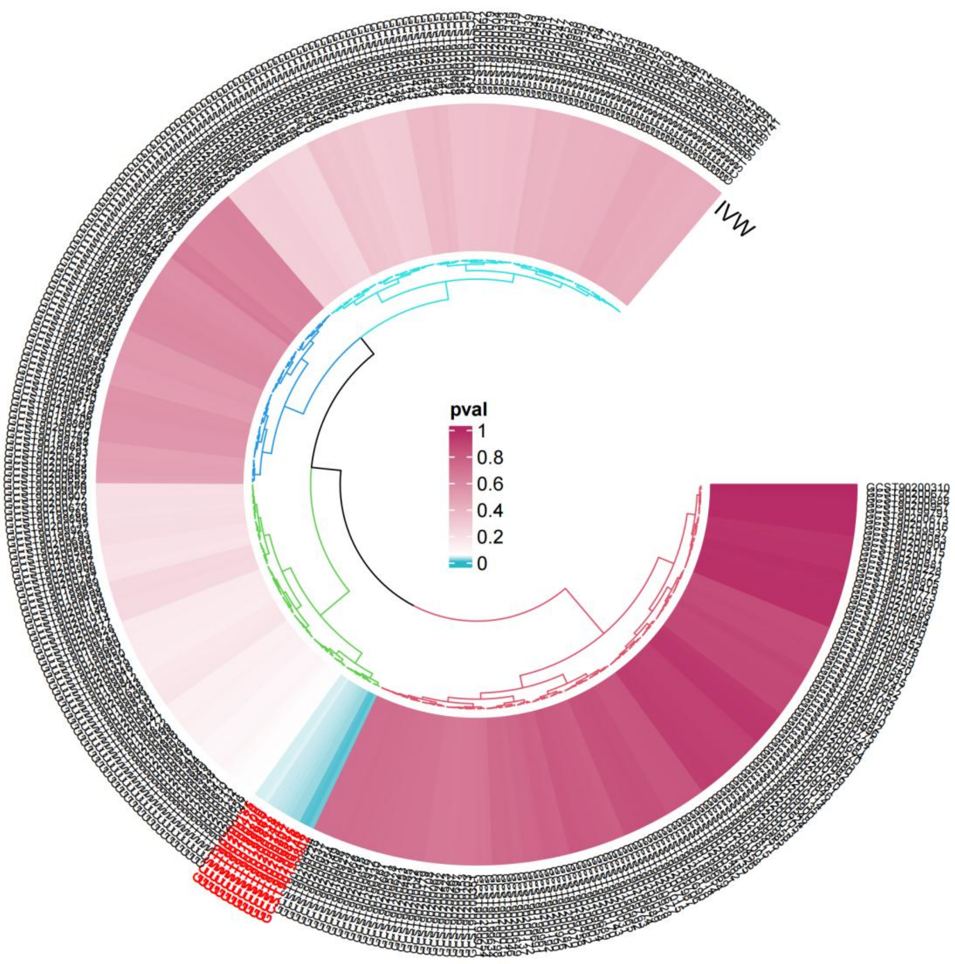

Supplement4 S2|Circular heat map of suggestive genetic correlation between Serum metabolites and(A)Lymphoid leukaemia (B)ALL (C)CLL.

A

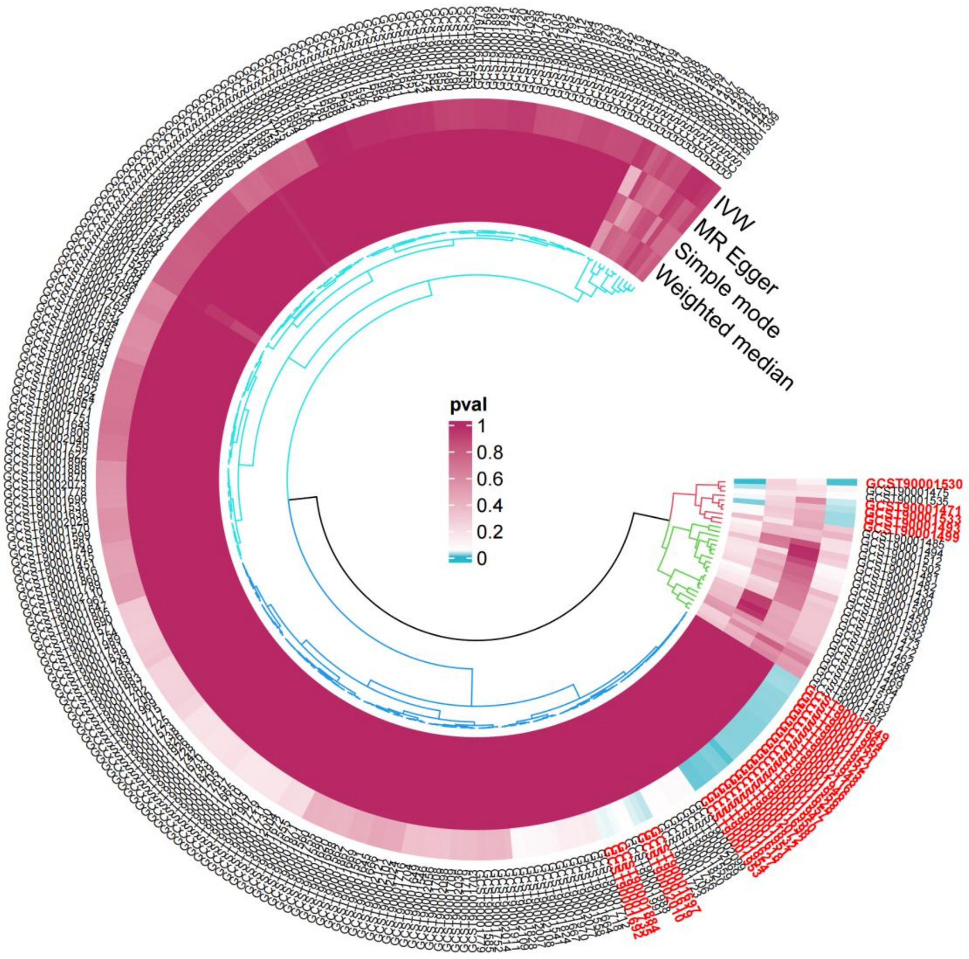
IVW
MR Egger
Simple mode
Weighted median
pval
1
0.8
0.6
0.4
0.2
0
GCST90001530

B

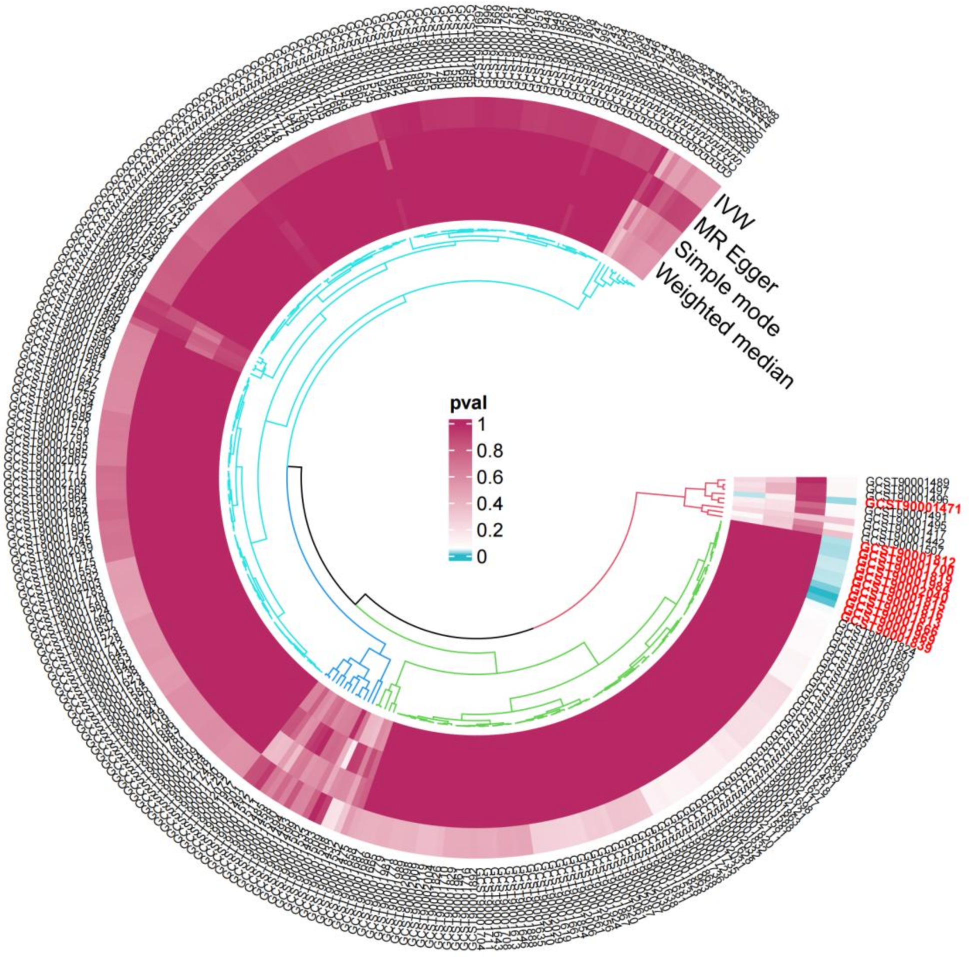
IVW
MR Egger
Simple mode
Weighted median
pval
1
0.8
0.6
0.4
0.2
0
GCST90001489
GCST90001471

C

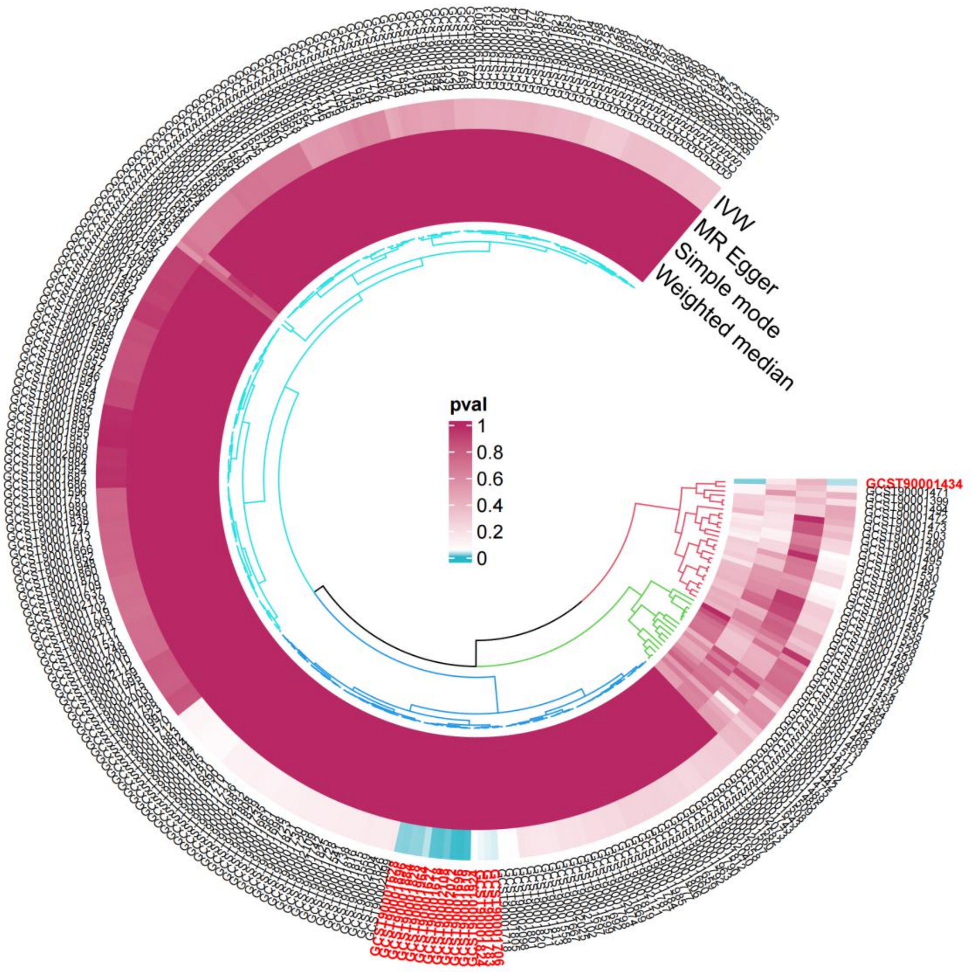

Supplement4 S3|Circular heat map of suggestive genetic correlation between Immune cells and(A)Lymphoid leukaemia (B)ALL (C)CLL.
