# Supplementary material for: Causal pathways in lymphoid leukemia: the gut microbiota, immune cells, and serum metabolites
Source: Front Immunol. 2024 Sep 16;15:1437869. doi: 10.3389/fimmu.2024.1437869 (PMC11439652; doi:10.3389/fimmu.2024.1437869)
Supplement: Supplementary file 1 [file DataSheet1.zip › Supplementary Figure 6.pdf]

S1

exposure	outcome	nsnp	method	pval	OR(95% CI)
CD62L− plasmacytoid Dendritic Cell %Dendritic Cell	1−palmitoyl−GPE (16:0)	19	MR Egger	**0.024**	1.070 (1.014 to 1.128)
		19	Weighted median	0.073	1.050 (0.995 to 1.107)
		19	Inverse variance weighted	**<0.001**	1.069 (1.030 to 1.109)
		19	Simple mode	0.189	1.057 (0.976 to 1.145)
		19	Weighted mode	0.142	1.046 (0.987 to 1.109)
1−palmitoyl−GPE (16:0)	Lymphoid leukaemia	6	MR Egger	0.803	0.868 (0.305 to 2.466)
		6	Weighted median	0.163	1.271 (0.907 to 1.781)
		6	Inverse variance weighted	**0.021**	1.350 (1.046 to 1.742)
		6	Simple mode	0.177	1.507 (0.903 to 2.513)
		6	Weighted mode	0.591	1.130 (0.746 to 1.712)
CD62L− plasmacytoid Dendritic Cell %Dendritic Cell	Lymphoid leukaemia	3	MR Egger	0.604	285.146 (0.000 to 1.440*10^{9}
		3	Weighted median	0.057	0.794 (0.626 to 1.010)
		3	Inverse variance weighted	**0.035**	0.787 (0.631 to 0.983)
		3	Simple mode	0.221	0.788 (0.604 to 1.030)
		3	Weighted mode	0.199	0.795 (0.626 to 1.010)

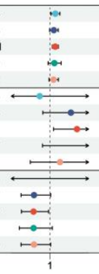

S2

exposure	outcome	nsnp	method	pval	OR(95% CI)
CD62L− plasmacytoid Dendritic Cell %Dendritic Cell	X−25371	19	MR Egger	**0.041**	0.941 (0.892 to 0.993)
		19	Weighted median	**0.037**	0.945 (0.896 to 0.997)
		19	Inverse variance weighted	**0.010**	0.953 (0.919 to 0.989)
		19	Simple mode	**0.032**	0.914 (0.847 to 0.986)
		19	Weighted mode	0.067	0.945 (0.893 to 1.000)
X−25371	Lymphoid leukaemia	4	MR Egger	0.738	1.117 (0.634 to 1.969)
		4	Weighted median	**0.023**	1.349 (1.041 to 1.747)
		4	Inverse variance weighted	**0.010**	1.379 (1.081 to 1.758)
		4	Simple mode	0.295	1.306 (0.865 to 1.971)
		4	Weighted mode	0.210	1.293 (0.941 to 1.776)
CD62L− plasmacytoid Dendritic Cell %Dendritic Cell	Lymphoid leukaemia	3	MR Egger	0.604	285.146 (0.000 to 1.440*10^{9})
		3	Weighted median	0.057	0.794 (0.626 to 1.010)
		3	Inverse variance weighted	**0.035**	0.787 (0.631 to 0.983)
		3	Simple mode	0.221	0.788 (0.604 to 1.030)
		3	Weighted mode	0.199	0.795 (0.626 to 1.010)

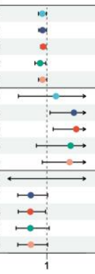

S3

exposure	outcome	nsnp	method	pval	OR(95% CI)
HLA DR+ T cell%T cell	1−palmitoyl−GPE (16:0)	31	MR Egger	0.331	1.016 (0.984 to 1.049)
		31	Weighted median	0.324	1.018 (0.983 to 1.054)
		31	Inverse variance weighted	**0.028**	1.028 (1.003 to 1.053)
		31	Simple mode	0.387	1.023 (0.973 to 1.075)
		31	Weighted mode	0.314	1.017 (0.985 to 1.051)
1−palmitoyl−GPE (16:0)	Lymphoid leukaemia	6	MR Egger	0.803	0.868 (0.305 to 2.466)
		6	Weighted median	0.163	1.271 (0.907 to 1.781)
		6	Inverse variance weighted	**0.021**	1.350 (1.046 to 1.742)
		6	Simple mode	0.177	1.507 (0.903 to 2.513)
		6	Weighted mode	0.591	1.130 (0.746 to 1.712)
HLA DR+ T cell%T cell	Lymphoid leukaemia	4	MR Egger	0.267	0.794 (0.590 to 1.067)
		4	Weighted median	**0.021**	0.838 (0.722 to 0.972)
		4	Inverse variance weighted	**0.037**	0.851 (0.732 to 0.990)
		4	Simple mode	0.249	0.832 (0.646 to 1.071)
		4	Weighted mode	0.132	0.834 (0.702 to 0.992)

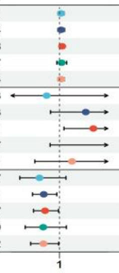

S4

exposure	outcome	nsnp	method	pval	OR(95% CI)
HLA DR+ T cell%T cell	Androstenediol (3alpha, 17alpha) monosulfate (3)	31	MR Egger	0.155	0.979 (0.952 to 1.007)
		31	Weighted median	0.180	0.978 (0.947 to 1.010)
		31	Inverse variance weighted	**0.036**	0.977 (0.956 to 0.999)
		31	Simple mode	0.134	0.958 (0.908 to 1.012)
		31	Weighted mode	0.076	0.970 (0.939 to 1.002)
Androstenediol (3alpha, 17alpha) monosulfate (3)	Lymphoid leukaemia	7	MR Egger	0.263	1.174 (0.917 to 1.502)
		7	Weighted median	**0.018**	1.185 (1.029 to 1.365)
		7	Inverse variance weighted	**0.014**	1.209 (1.038 to 1.409)
		7	Simple mode	**0.049**	1.449 (1.078 to 1.948)
		7	Weighted mode	0.092	1.175 (1.004 to 1.374)
HLA DR+ T cell%T cell	Lymphoid leukaemia	4	MR Egger	0.267	0.794 (0.590 to 1.067)
		4	Weighted median	**0.021**	0.838 (0.722 to 0.972)
		4	Inverse variance weighted	**0.037**	0.851 (0.732 to 0.990)
		4	Simple mode	0.249	0.832 (0.646 to 1.071)
		4	Weighted mode	0.132	0.834 (0.702 to 0.992)

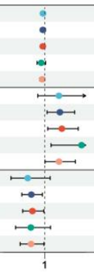

S5

exposure	outcome	nsnp	method	pval	OR(95% CI)
HLA DR+ T cell%T cell	1−stearoyl−2−linoleoyl−GPI (18:0/18:2)	31	MR Egger	0.389	1.016 (0.980 to 1.054)
		31	Weighted median	0.199	1.023 (0.988 to 1.059)
		31	Inverse variance weighted	**0.045**	1.029 (1.001 to 1.058)
		31	Simple mode	0.495	1.020 (0.964 to 1.081)
		31	Weighted mode	0.142	1.023 (0.993 to 1.054)
1−stearoyl−2−linoleoyl−GPI (18:0/18:2)	Lymphoid leukaemia	4	MR Egger	0.619	1.779 (0.257 to 12.335)
		4	Weighted median	0.096	0.814 (0.638 to 1.038)
		4	Inverse variance weighted	**0.040**	0.796 (0.640 to 0.990)
		4	Simple mode	0.456	0.870 (0.632 to 1.198)
		4	Weighted mode	0.316	0.835 (0.623 to 1.121)
HLA DR+ T cell%T cell	Lymphoid leukaemia	4	MR Egger	0.267	0.794 (0.590 to 1.067)
		4	Weighted median	**0.021**	0.838 (0.722 to 0.972)
		4	Inverse variance weighted	**0.037**	0.851 (0.732 to 0.990)
		4	Simple mode	0.249	0.832 (0.646 to 1.071)
		4	Weighted mode	0.132	0.834 (0.702 to 0.992)

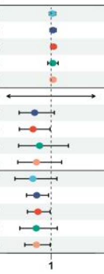

S6

exposure	outcome	nsnp	method	pval	OR(95% CI)
HLA DR+ T cell%T cell	Aspartate to N−acetylglucosamine to N−acetylgalactosamine ratio	31	MR Egger	**0.008**	0.955 (0.925 to 0.986)
		31	Weighted median	0.060	0.963 (0.926 to 1.002)
		31	Inverse variance weighted	**0.015**	0.969 (0.945 to 0.994)
		31	Simple mode	0.978	0.999 (0.932 to 1.071)
		31	Weighted mode	**0.035**	0.960 (0.926 to 0.995)
Aspartate to N−acetylglucosamine to N−acetylgalactosamine ratio	Lymphoid leukaemia	3	MR Egger	0.290	0.412 (0.176 to 0.965)
		3	Weighted median	**0.008**	0.666 (0.493 to 0.901)
		3	Inverse variance weighted	**0.007**	0.678 (0.512 to 0.900)
		3	Simple mode	0.262	0.705 (0.453 to 1.099)
		3	Weighted mode	0.124	0.636 (0.450 to 0.898)
HLA DR+ T cell%T cell	Lymphoid leukaemia	4	MR Egger	0.267	0.794 (0.590 to 1.067)
		4	Weighted median	**0.021**	0.838 (0.722 to 0.972)
		4	Inverse variance weighted	**0.037**	0.851 (0.732 to 0.990)
		4	Simple mode	0.249	0.832 (0.646 to 1.071)
		4	Weighted mode	0.132	0.834 (0.702 to 0.992)

S7

exposure	outcome	nsnp	method	pval	OR(95% CI)
HLA DR+ CD8+ T cell Absolute Count	N6,n6,n6−trimethyllysine	32	MR Egger	0.150	1.030 (0.990 to 1.070)
		32	Weighted median	0.063	1.037 (0.998 to 1.078)
		32	Inverse variance weighted	**0.021**	1.032 (1.005 to 1.060)
		32	Simple mode	0.159	1.049 (0.983 to 1.118)
		32	Weighted mode	0.132	1.031 (0.992 to 1.072)
N6,n6,n6−trimethyllysine	Lymphoid leukaemia	3	MR Egger	0.454	0.760 (0.476 to 1.211)
		3	Weighted median	**0.013**	0.689 (0.514 to 0.925)
		3	Inverse variance weighted	**0.008**	0.684 (0.518 to 0.903)
		3	Simple mode	0.270	0.715 (0.462 to 1.106)
		3	Weighted mode	0.155	0.707 (0.522 to 0.958)
HLA DR+ CD8+ T cell Absolute Count	Lymphoid leukaemia	3	MR Egger	0.626	0.878 (0.599 to 1.287)
		3	Weighted median	**0.020**	0.806 (0.671 to 0.967)
		3	Inverse variance weighted	**0.012**	0.802 (0.675 to 0.953)
		3	Simple mode	0.154	0.723 (0.545 to 0.959)
		3	Weighted mode	0.177	0.818 (0.675 to 0.991)

S8

exposure	outcome	nsnp	method	pval	OR(95% CI)
HLA DR+ CD8+ T cell Absolute Count	Aspartate to N−acetylglucosamine to N−acetylgalactosamine ratio	32	MR Egger	0.197	0.972 (0.933 to 1.014)
		32	Weighted median	0.427	0.984 (0.945 to 1.024)
		32	Inverse variance weighted	**0.024**	0.968 (0.941 to 0.996)
		32	Simple mode	0.499	0.976 (0.909 to 1.047)
		32	Weighted mode	0.294	0.981 (0.946 to 1.017)
Aspartate to N−acetylglucosamine to N−acetylgalactosamine ratio	Lymphoid leukaemia	3	MR Egger	0.290	0.412 (0.176 to 0.965)
		3	Weighted median	**0.008**	0.666 (0.493 to 0.901)
		3	Inverse variance weighted	**0.007**	0.678 (0.512 to 0.900)
		3	Simple mode	0.262	0.705 (0.453 to 1.099)
		3	Weighted mode	0.124	0.636 (0.450 to 0.898)
HLA DR+ CD8+ T cell Absolute Count	Lymphoid leukaemia	3	MR Egger	0.626	0.878 (0.599 to 1.287)
		3	Weighted median	**0.020**	0.806 (0.671 to 0.967)
		3	Inverse variance weighted	**0.012**	0.802 (0.675 to 0.953)
		3	Simple mode	0.154	0.723 (0.545 to 0.959)
		3	Weighted mode	0.177	0.818 (0.675 to 0.991)

S9

exposure	outcome	nsnp	method	pval	OR(95% CI)
HLA DR+ CD8+ T cell %T celll	X−24588	30	MR Egger	**0.010**	1.048 (1.013 to 1.083)
		30	Weighted median	0.156	1.031 (0.989 to 1.075)
		30	Inverse variance weighted	**0.016**	1.032 (1.006 to 1.058)
		30	Simple mode	0.994	1.000 (0.929 to 1.077)
		30	Weighted mode	0.118	1.033 (0.993 to 1.074)
X−24588	Lymphoid leukaemia	6	MR Egger	0.148	0.463 (0.200 to 1.077)
		6	Weighted median	**0.032**	0.610 (0.388 to 0.957)
		6	Inverse variance weighted	**0.039**	0.644 (0.424 to 0.978)
		6	Simple mode	0.215	0.578 (0.270 to 1.233)
		6	Weighted mode	0.238	0.624 (0.313 to 1.243)
HLA DR+ CD8+ T cell %T cell	Lymphoid leukaemia	3	MR Egger	0.574	0.875 (0.627 to 1.220)
		3	Weighted median	**0.029**	0.820 (0.688 to 0.979)
		3	Inverse variance weighted	**0.017**	0.815 (0.688 to 0.964)
		3	Simple mode	0.260	0.782 (0.574 to 1.066)
		3	Weighted mode	0.155	0.825 (0.697 to 0.977)

S10

exposure	outcome	nsnp	method	pval	OR(95% CI)
HLA DR+ CD8+ T cell %T cell	X−25371	30	MR Egger	**0.042**	1.037 (1.003 to 1.072)
		30	Weighted median	**0.043**	1.040 (1.001 to 1.080)
		30	Inverse variance weighted	**0.029**	1.028 (1.003 to 1.054)
		30	Simple mode	0.395	1.030 (0.964 to 1.100)
		30	Weighted mode	**0.029**	1.042 (1.006 to 1.078)
X−25371	Lymphoid leukaemia	4	MR Egger	0.738	1.117 (0.634 to 1.969)
		4	Weighted median	**0.023**	1.349 (1.041 to 1.747)
		4	Inverse variance weighted	**0.010**	1.379 (1.081 to 1.758)
		4	Simple mode	0.295	1.306 (0.865 to 1.971)
		4	Weighted mode	0.210	1.293 (0.941 to 1.776)
HLA DR+ CD8+ T cell %T cell	Lymphoid leukaemia	3	MR Egger	0.574	0.875 (0.627 to 1.220)
		3	Weighted median	**0.029**	0.820 (0.688 to 0.979)
		3	Inverse variance weighted	**0.017**	0.815 (0.688 to 0.964)
		3	Simple mode	0.260	0.782 (0.574 to 1.066)
		3	Weighted mode	0.155	0.825 (0.697 to 0.977)

S11

exposure	outcome	nsnp	method	pval	OR(95% CI)
CD45RA− CD28− CD8+ T cell %T cell	1−stearoyl−2−linoleoyl−GPI (18:0/18:2)	178	MR Egger	0.249	1.001 (1.000 to 1.001)
		178	Weighted median	**0.039**	1.001 (1.000 to 1.002)
		178	Inverse variance weighted	**0.029**	1.001 (1.000 to 1.001)
		178	Simple mode	0.103	1.001 (1.000 to 1.003)
		178	Weighted mode	**0.036**	1.001 (1.000 to 1.002)
1−stearoyl−2−linoleoyl−GPI (18:0/18:2)	Lymphoid leukaemia	4	MR Egger	0.619	1.779 (0.257 to 12.335)
		4	Weighted median	0.096	0.814 (0.638 to 1.038)
		4	Inverse variance weighted	**0.040**	0.796 (0.640 to 0.990)
		4	Simple mode	0.456	0.870 (0.632 to 1.198)
		4	Weighted mode	0.316	0.835 (0.623 to 1.121)
CD45RA− CD28− CD8+ T cell %T cell	Lymphoid leukaemia	35	MR Egger	**0.023**	1.003 (1.001 to 1.005)
		35	Weighted median	0.103	1.002 (1.000 to 1.004)
		35	Inverse variance weighted	**0.031**	1.002 (1.000 to 1.004)
		35	Simple mode	0.638	1.001 (0.997 to 1.005)
		35	Weighted mode	0.070	1.002 (1.000 to 1.004)

S12

exposure	outcome	nsnp	method	pval	OR(95% CI)
CD45RA+ CD28− CD8+ T cell Absolute Count	Deoxycholic acid glucuronide	715	MR Egger	0.112	1.000 (1.000 to 1.000)
		715	Weighted median	0.328	1.000 (1.000 to 1.000)
		715	Inverse variance weighted	**0.017**	1.000 (1.000 to 1.000)
		715	Simple mode	0.341	1.000 (1.000 to 1.000)
		715	Weighted mode	0.664	1.000 (1.000 to 1.000)
Deoxycholic acid glucuronide	Lymphoid leukaemia	3	MR Egger	0.578	1.402 (0.600 to 3.276)
		3	Weighted median	0.053	1.221 (0.996 to 1.498)
		3	Inverse variance weighted	**0.036**	1.219 (1.014 to 1.466)
		3	Simple mode	0.297	1.212 (0.926 to 1.585)
		3	Weighted mode	0.222	1.219 (0.977 to 1.521)
CD45RA+ CD28− CD8+ T cell Absolute Count	Lymphoid leukaemia	16	MR Egger	0.516	1.002 (0.998 to 1.006)
		16	Weighted median	0.132	1.001 (0.999 to 1.003)
		16	Inverse variance weighted	**0.018**	1.001 (1.001 to 1.001)
		16	Simple mode	0.243	1.001 (0.999 to 1.003)
		16	Weighted mode	0.078	1.001 (0.999 to 1.003)

S13

exposure	outcome	nsnp	method	pval	OR(95% CI)
IgD on IgD+ CD24− B cell	1−palmitoyl−GPE (16:0)	25	MR Egger	0.552	1.028 (0.940 to 1.124)
		25	Weighted median	0.167	1.045 (0.982 to 1.113)
		25	Inverse variance weighted	**0.028**	1.051 (1.005 to 1.099)
		25	Simple mode	0.386	1.049 (0.944 to 1.166)
		25	Weighted mode	0.325	1.045 (0.959 to 1.137)
1−palmitoyl−GPE (16:0)	Lymphoid leukaemia	6	MR Egger	0.803	0.868 (0.305 to 2.466)
		6	Weighted median	0.163	1.271 (0.907 to 1.781)
		6	Inverse variance weighted	**0.021**	1.350 (1.046 to 1.742)
		6	Simple mode	0.177	1.507 (0.903 to 2.513)
		6	Weighted mode	0.591	1.130 (0.746 to 1.712)
IgD on IgD+ CD24− B cell	Lymphoid leukaemia	5	MR Egger	0.426	0.914 (0.754 to 1.107)
		5	Weighted median	**0.017**	0.874 (0.781 to 0.977)
		5	Inverse variance weighted	**0.020**	0.891 (0.810 to 0.981)
		5	Simple mode	0.162	0.831 (0.673 to 1.027)
		5	Weighted mode	0.094	0.864 (0.758 to 0.985)

S14

exposure	outcome	nsnp	method	pval	OR(95% CI)
CD33 on CD14+ monocyte	DHEAS	21	MR Egger	0.262	0.981 (0.950 to 1.013)
		21	Weighted median	**0.016**	0.971 (0.948 to 0.995)
		21	Inverse variance weighted	**0.018**	0.976 (0.957 to 0.996)
		21	Simple mode	0.662	0.986 (0.928 to 1.049)
		21	Weighted mode	**0.022**	0.970 (0.947 to 0.994)
DHEAS	Lymphoid leukaemia	3	MR Egger	0.596	1.183 (0.755 to 1.853)
		3	Weighted median	0.059	1.318 (0.990 to 1.754)
		3	Inverse variance weighted	**0.042**	1.342 (1.010 to 1.783)
		3	Simple mode	0.299	1.418 (0.867 to 2.319)
		3	Weighted mode	0.235	1.298 (0.956 to 1.763)
CD33 on CD14+ monocyte	Lymphoid leukaemia	6	MR Egger	0.108	1.112 (1.006 to 1.229)
		6	Weighted median	**0.016**	1.080 (1.014 to 1.150)
		6	Inverse variance weighted	**0.017**	1.076 (1.012 to 1.143)
		6	Simple mode	0.399	1.060 (0.937 to 1.199)
		6	Weighted mode	0.065	1.080 (1.014 to 1.150)

S15

exposure	outcome	nsnp	method	pval	OR(95% CI)
CD33 on CD14+ monocyte	Androstenediol (3beta,17beta) disulfate (1)	21	MR Egger	0.638	0.992 (0.960 to 1.025)
		21	Weighted median	0.060	0.977 (0.954 to 1.001)
		21	Inverse variance weighted	**0.020**	0.975 (0.955 to 0.996)
		21	Simple mode	0.440	0.976 (0.918 to 1.037)
		21	Weighted mode	0.093	0.978 (0.955 to 1.002)
Androstenediol (3beta,17beta) disulfate (1)	Lymphoid leukaemia	3	MR Egger	0.379	1.402 (0.895 to 2.196)
		3	Weighted median	**<0.001**	1.513 (1.215 to 1.884)
		3	Inverse variance weighted	**<0.001**	1.525 (1.234 to 1.885)
		3	Simple mode	0.119	1.498 (1.108 to 2.026)
		3	Weighted mode	0.087	1.511 (1.171 to 1.950)
CD33 on CD14+ monocyte	Lymphoid leukaemia	6	MR Egger	0.108	1.112 (1.006 to 1.229)
		6	Weighted median	**0.016**	1.080 (1.014 to 1.150)
		6	Inverse variance weighted	**0.017**	1.076 (1.012 to 1.143)
		6	Simple mode	0.399	1.060 (0.937 to 1.199)
		6	Weighted mode	0.065	1.080 (1.014 to 1.150)

S16

exposure	outcome	nsnp	method	pval	OR(95% CI)
CD33 on CD33dim HLA DR+ CD11b+	DHEAS	22	MR Egger	0.060	0.968 (0.938 to 0.999)
		22	Weighted median	**0.012**	0.969 (0.946 to 0.993)
		22	Inverse variance weighted	**0.049**	0.980 (0.961 to 1.000)
		22	Simple mode	0.632	0.985 (0.927 to 1.047)
		22	Weighted mode	**0.019**	0.970 (0.947 to 0.993)
DHEAS	Lymphoid leukaemia	3	MR Egger	0.596	1.183 (0.755 to 1.853)
		3	Weighted median	0.059	1.318 (0.990 to 1.754)
		3	Inverse variance weighted	**0.042**	1.342 (1.010 to 1.783)
		3	Simple mode	0.299	1.418 (0.867 to 2.319)
		3	Weighted mode	0.235	1.298 (0.956 to 1.763)
CD33 on CD33dim HLA DR+ CD11b+	Lymphoid leukaemia	7	MR Egger	0.084	1.112 (1.010 to 1.224)
		7	Weighted median	**0.020**	1.077 (1.011 to 1.147)
		7	Inverse variance weighted	**0.018**	1.074 (1.012 to 1.139)
		7	Simple mode	0.542	1.042 (0.919 to 1.181)
		7	Weighted mode	0.065	1.078 (1.010 to 1.150)

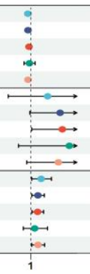

S17

exposure	outcome	nsnp	method	pval	OR(95% CI)
CD33 on CD33dim HLA DR+ CD11b+	Androstenediol (3beta,17beta) monosulfate (1)	22	MR Egger	**0.037**	0.964 (0.934 to 0.996)
		22	Weighted median	**0.016**	0.971 (0.948 to 0.995)
		22	Inverse variance weighted	**0.045**	0.980 (0.961 to 1.000)
		22	Simple mode	0.879	0.995 (0.940 to 1.055)
		22	Weighted mode	**0.024**	0.972 (0.950 to 0.995)
Androstenediol (3beta,17beta) monosulfate (1)	Lymphoid leukaemia	3	MR Egger	0.528	1.283 (0.754 to 2.182)
		3	Weighted median	**0.014**	1.465 (1.081 to 1.985)
		3	Inverse variance weighted	**0.008**	1.471 (1.103 to 1.962)
		3	Simple mode	0.144	1.910 (1.112 to 3.280)
		3	Weighted mode	0.246	1.328 (0.943 to 1.872)
CD33 on CD33dim HLA DR+ CD11b+	Lymphoid leukaemia	7	MR Egger	0.084	1.112 (1.010 to 1.224)
		7	Weighted median	**0.020**	1.077 (1.011 to 1.147)
		7	Inverse variance weighted	**0.018**	1.074 (1.012 to 1.139)
		7	Simple mode	0.542	1.042 (0.919 to 1.181)
		7	Weighted mode	0.065	1.078 (1.010 to 1.150)

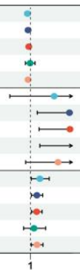

S18

exposure	outcome	nsnp	method	pval	OR(95% CI)
CD33 on CD33dim HLA DR+ CD11b+	Aspartate to N−acetylglucosamine to N−acetylgalactosamine ratio	22	MR Egger	0.349	1.017 (0.982 to 1.053)
		22	Weighted median	0.105	1.022 (0.996 to 1.049)
		22	Inverse variance weighted	**0.025**	1.025 (1.003 to 1.048)
		22	Simple mode	0.852	1.005 (0.950 to 1.064)
		22	Weighted mode	0.197	1.018 (0.992 to 1.044)
Aspartate to N−acetylglucosamine to N−acetylgalactosamine ratio	Lymphoid leukaemia	3	MR Egger	0.290	0.412 (0.176 to 0.965)
		3	Weighted median	**0.008**	0.666 (0.493 to 0.901)
		3	Inverse variance weighted	**0.007**	0.678 (0.512 to 0.900)
		3	Simple mode	0.262	0.705 (0.453 to 1.099)
		3	Weighted mode	0.124	0.636 (0.450 to 0.898)
CD33 on CD33dim HLA DR+ CD11b+	Lymphoid leukaemia	7	MR Egger	0.084	1.112 (1.010 to 1.224)
		7	Weighted median	**0.020**	1.077 (1.011 to 1.147)
		7	Inverse variance weighted	**0.018**	1.074 (1.012 to 1.139)
		7	Simple mode	0.542	1.042 (0.919 to 1.181)
		7	Weighted mode	0.065	1.078 (1.010 to 1.150)

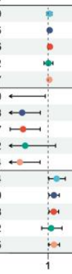

S19

exposure	outcome	nsnp	method	pval	OR(95% CI)
CD33 on Granulocytic Myeloid−Derived Suppressor Cells	DHEAS	19	MR Egger	**0.034**	0.952 (0.913 to 0.993)
		19	Weighted median	**0.014**	0.961 (0.932 to 0.992)
		19	Inverse variance weighted	**0.003**	0.967 (0.946 to 0.989)
		19	Simple mode	0.327	0.972 (0.920 to 1.027)
		19	Weighted mode	**0.033**	0.961 (0.929 to 0.994)
DHEAS	Lymphoid leukaemia	3	MR Egger	0.596	1.183 (0.755 to 1.853)
		3	Weighted median	0.059	1.318 (0.990 to 1.754)
		3	Inverse variance weighted	**0.042**	1.342 (1.010 to 1.783)
		3	Simple mode	0.299	1.418 (0.867 to 2.319)
		3	Weighted mode	0.235	1.298 (0.956 to 1.763)
CD33 on Granulocytic Myeloid−Derived Suppressor Cells	Lymphoid leukaemia	3	MR Egger	0.314	1.165 (0.992 to 1.369)
		3	Weighted median	**0.017**	1.115 (1.019 to 1.220)
		3	Inverse variance weighted	**0.017**	1.107 (1.018 to 1.205)
		3	Simple mode	0.218	1.139 (0.987 to 1.314)
		3	Weighted mode	0.129	1.120 (1.025 to 1.223)

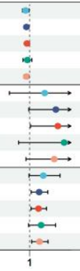

S20

exposure	outcome	nsnp	method	pval	OR(95% CI)
CD33 on Granulocytic Myeloid−Derived Suppressor Cells	Androstenediol (3beta,17beta) monosulfate (1)	19	MR Egger	**0.024**	0.948 (0.909 to 0.989)
		19	Weighted median	**0.015**	0.962 (0.933 to 0.992)
		19	Inverse variance weighted	**0.004**	0.968 (0.947 to 0.990)
		19	Simple mode	0.248	0.964 (0.908 to 1.024)
		19	Weighted mode	**0.021**	0.962 (0.933 to 0.991)
Androstenediol (3beta,17beta) monosulfate (1)	Lymphoid leukaemia	3	MR Egger	0.528	1.283 (0.754 to 2.182)
		3	Weighted median	**0.014**	1.465 (1.081 to 1.985)
		3	Inverse variance weighted	**0.008**	1.471 (1.103 to 1.962)
		3	Simple mode	0.144	1.910 (1.112 to 3.280)
		3	Weighted mode	0.246	1.328 (0.943 to 1.872)
CD33 on Granulocytic Myeloid−Derived Suppressor Cells	Lymphoid leukaemia	3	MR Egger	0.314	1.165 (0.992 to 1.369)
		3	Weighted median	**0.017**	1.115 (1.019 to 1.220)
		3	Inverse variance weighted	**0.017**	1.107 (1.018 to 1.205)
		3	Simple mode	0.218	1.139 (0.987 to 1.314)
		3	Weighted mode	0.129	1.120 (1.025 to 1.223)

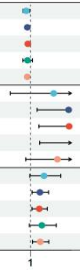

S21

exposure	outcome	nsnp	method	pval	OR(95% CI)
CD33 on CD66b++ myeloid cell	DHEAS	19	MR Egger	0.141	0.969 (0.932 to 1.008)
		19	Weighted median	**0.010**	0.964 (0.938 to 0.991)
		19	Inverse variance weighted	**0.033**	0.977 (0.957 to 0.998)
		19	Simple mode	0.426	0.975 (0.916 to 1.037)
		19	Weighted mode	**0.031**	0.963 (0.933 to 0.994)
DHEAS	Lymphoid leukaemia	3	MR Egger	0.596	1.183 (0.755 to 1.853)
		3	Weighted median	0.059	1.318 (0.990 to 1.754)
		3	Inverse variance weighted	**0.042**	1.342 (1.010 to 1.783)
		3	Simple mode	0.299	1.418 (0.867 to 2.319)
		3	Weighted mode	0.235	1.298 (0.956 to 1.763)
CD33 on CD66b++ myeloid cell	Lymphoid leukaemia	5	MR Egger	0.169	1.127 (0.989 to 1.286)
		5	Weighted median	**0.019**	1.095 (1.015 to 1.182)
		5	Inverse variance weighted	**0.016**	1.090 (1.016 to 1.169)
		5	Simple mode	0.208	1.114 (0.967 to 1.283)
		5	Weighted mode	0.088	1.097 (1.013 to 1.189)

S22

exposure	outcome	nsnp	method	pval	OR(95% CI)
CD33 on CD66b++ myeloid cell	Androstenediol (3beta,17beta) monosulfate (1)	19	MR Egger	0.101	0.965 (0.928 to 1.005)
		19	Weighted median	**0.018**	0.969 (0.944 to 0.995)
		19	Inverse variance weighted	**0.015**	0.974 (0.954 to 0.995)
		19	Simple mode	0.718	0.990 (0.939 to 1.044)
		19	Weighted mode	**0.025**	0.965 (0.938 to 0.993)
Androstenediol (3beta,17beta) monosulfate (1)	Lymphoid leukaemia	3	MR Egger	0.528	1.283 (0.754 to 2.182)
		3	Weighted median	**0.014**	1.465 (1.081 to 1.985)
		3	Inverse variance weighted	**0.008**	1.471 (1.103 to 1.962)
		3	Simple mode	0.144	1.910 (1.112 to 3.280)
		3	Weighted mode	0.246	1.328 (0.943 to 1.872)
CD33 on CD66b++ myeloid cell	Lymphoid leukaemia	5	MR Egger	0.169	1.127 (0.989 to 1.286)
		5	Weighted median	**0.019**	1.095 (1.015 to 1.182)
		5	Inverse variance weighted	**0.016**	1.090 (1.016 to 1.169)
		5	Simple mode	0.208	1.114 (0.967 to 1.283)
		5	Weighted mode	0.088	1.097 (1.013 to 1.189)

S23

exposure	outcome	nsnp	method	pval	OR(95% CI)
CD33 on CD66b++ myeloid cell	Aspartate to N-acetylglucosamine to N-acetylgalactosamine ratio	19	MR Egger	0.069	1.044 (1.000 to 1.090)
		19	Weighted median	0.098	1.026 (0.995 to 1.058)
		19	Inverse variance weighted	**0.024**	1.027 (1.003 to 1.051)
		19	Simple mode	0.376	1.025 (0.972 to 1.082)
		19	Weighted mode	0.117	1.025 (0.995 to 1.056)
Aspartate to N-acetylglucosamine to N-acetylgalactosamine ratio	Lymphoid leukaemia	3	MR Egger	0.290	0.412 (0.176 to 0.965)
		3	Weighted median	**0.008**	0.666 (0.493 to 0.901)
		3	Inverse variance weighted	**0.007**	0.678 (0.512 to 0.900)
		3	Simple mode	0.262	0.705 (0.453 to 1.099)
		3	Weighted mode	0.124	0.636 (0.450 to 0.898)
CD33 on CD66b++ myeloid cell	Lymphoid leukaemia	5	MR Egger	0.169	1.127 (0.989 to 1.286)
		5	Weighted median	**0.019**	1.095 (1.015 to 1.182)
		5	Inverse variance weighted	**0.016**	1.090 (1.016 to 1.169)
		5	Simple mode	0.208	1.114 (0.967 to 1.283)
		5	Weighted mode	0.088	1.097 (1.013 to 1.189)

S24

exposure	outcome	nsnp	method	pval	OR(95% CI)
CD33 on Monocytic Myeloid-Derived Suppressor Cells	DHEAS	20	MR Egger	0.188	0.974 (0.937 to 1.012)
		20	Weighted median	**0.012**	0.970 (0.947 to 0.993)
		20	Inverse variance weighted	0.050	0.976 (0.953 to 1.000)
		20	Simple mode	0.781	0.991 (0.933 to 1.053)
		20	Weighted mode	**0.019**	0.970 (0.948 to 0.993)
DHEAS	Lymphoid leukaemia	3	MR Egger	0.596	1.183 (0.755 to 1.853)
		3	Weighted median	0.059	1.318 (0.990 to 1.754)
		3	Inverse variance weighted	**0.042**	1.342 (1.010 to 1.783)
		3	Simple mode	0.299	1.418 (0.867 to 2.319)
		3	Weighted mode	0.235	1.298 (0.956 to 1.763)
CD33 on Monocytic Myeloid-Derived Suppressor Cells	Lymphoid leukaemia	3	MR Egger	0.351	1.097 (0.981 to 1.227)
		3	Weighted median	**0.013**	1.080 (1.016 to 1.148)
		3	Inverse variance weighted	**0.019**	1.077 (1.011 to 1.147)
		3	Simple mode	0.218	1.097 (0.991 to 1.215)
		3	Weighted mode	0.148	1.080 (1.010 to 1.154)

S25

exposure	outcome	nsnp	method	pval	OR(95% CI)
CD33 on Monocytic Myeloid-Derived Suppressor Cells	Pregnenolone sulfate	20	MR Egger	0.493	0.988 (0.957 to 1.021)
		20	Weighted median	0.145	0.981 (0.957 to 1.006)
		20	Inverse variance weighted	**0.028**	0.976 (0.956 to 0.997)
		20	Simple mode	0.056	0.928 (0.864 to 0.997)
		20	Weighted mode	0.232	0.984 (0.958 to 1.010)
Pregnenolone sulfate	Lymphoid leukaemia	6	MR Egger	0.713	1.117 (0.643 to 1.942)
		6	Weighted median	0.086	1.336 (0.960 to 1.861)
		6	Inverse variance weighted	**0.038**	1.330 (1.017 to 1.739)
		6	Simple mode	0.318	1.278 (0.828 to 1.970)
		6	Weighted mode	0.215	1.327 (0.898 to 1.960)
CD33 on Monocytic Myeloid-Derived Suppressor Cells	Lymphoid leukaemia	3	MR Egger	0.351	1.097 (0.981 to 1.227)
		3	Weighted median	**0.013**	1.080 (1.016 to 1.148)
		3	Inverse variance weighted	**0.019**	1.077 (1.011 to 1.147)
		3	Simple mode	0.218	1.097 (0.991 to 1.215)
		3	Weighted mode	0.148	1.080 (1.010 to 1.154)

S26

exposure	outcome	nsnp	method	pval	OR(95% CI)
CD33 on CD33dim HLA DR−	Retinol (Vitamin A) to oleoyl−linoleoyl−glycerol (18:1 to 18:2) [2] ratio	20	MR Egger	**0.046**	0.963 (0.929 to 0.997)
		20	Weighted median	**0.008**	0.965 (0.939 to 0.991)
		20	Inverse variance weighted	**0.014**	0.973 (0.952 to 0.995)
		20	Simple mode	0.556	0.981 (0.922 to 1.044)
		20	Weighted mode	**0.009**	0.966 (0.943 to 0.989)
Retinol (Vitamin A) to oleoyl−linoleoyl−glycerol (18:1 to 18:2) [2] ratio	Lymphoid leukaemia	6	MR Egger	0.603	1.374 (0.456 to 4.143)
		6	Weighted median	0.090	1.443 (0.945 to 2.204)
		6	Inverse variance weighted	**0.020**	1.452 (1.059 to 1.991)
		6	Simple mode	0.172	1.642 (0.893 to 3.021)
		6	Weighted mode	0.413	1.324 (0.714 to 2.456)
CD33 on CD33dim HLA DR−	Lymphoid leukaemia	6	MR Egger	0.255	1.084 (0.962 to 1.222)
		6	Weighted median	**0.012**	1.087 (1.018 to 1.159)
		6	Inverse variance weighted	**0.011**	1.082 (1.018 to 1.150)
		6	Simple mode	0.153	1.129 (0.980 to 1.300)
		6	Weighted mode	0.066	1.081 (1.013 to 1.153)

S27

exposure	outcome	nsnp	method	pval	OR(95% CI)
CD33 on basophil	Aspartate to N−acetylglucosamine to N−acetylgalactosamine ratio	23	MR Egger	0.861	1.002 (0.983 to 1.021)
		23	Weighted median	0.481	1.007 (0.987 to 1.028)
		23	Inverse variance weighted	**0.034**	1.017 (1.001 to 1.033)
		23	Simple mode	0.427	1.015 (0.979 to 1.053)
		23	Weighted mode	0.262	1.009 (0.993 to 1.026)
Aspartate to N−acetylglucosamine to N−acetylgalactosamine ratio	Lymphoid leukaemia	3	MR Egger	0.290	0.412 (0.176 to 0.965)
		3	Weighted median	**0.008**	0.666 (0.493 to 0.901)
		3	Inverse variance weighted	**0.007**	0.678 (0.512 to 0.900)
		3	Simple mode	0.262	0.705 (0.453 to 1.099)
		3	Weighted mode	0.124	0.636 (0.450 to 0.898)
CD33 on basophil	Lymphoid leukaemia	7	MR Egger	0.241	1.112 (0.952 to 1.298)
		7	Weighted median	**0.008**	1.089 (1.023 to 1.159)
		7	Inverse variance weighted	**0.010**	1.087 (1.020 to 1.157)
		7	Simple mode	0.248	1.107 (0.947 to 1.295)
		7	Weighted mode	**0.047**	1.089 (1.019 to 1.164)

S28

exposure	outcome	nsnp	method	pval	OR(95% CI)
CD33 on Im MDSC	Retinol (Vitamin A) to oleoyl−linoleoyl−glycerol (18:1 to 18:2) [2] ratio	23	MR Egger	0.081	0.970 (0.938 to 1.002)
		23	Weighted median	**0.014**	0.967 (0.942 to 0.993)
		23	Inverse variance weighted	**0.010**	0.972 (0.952 to 0.993)
		23	Simple mode	0.460	0.977 (0.920 to 1.038)
		23	Weighted mode	**0.025**	0.968 (0.943 to 0.994)
Retinol (Vitamin A) to oleoyl−linoleoyl−glycerol (18:1 to 18:2) [2] ratio	Lymphoid leukaemia	6	MR Egger	0.603	1.374 (0.456 to 4.143)
		6	Weighted median	0.090	1.443 (0.945 to 2.204)
		6	Inverse variance weighted	**0.020**	1.452 (1.059 to 1.991)
		6	Simple mode	0.172	1.642 (0.893 to 3.021)
		6	Weighted mode	0.413	1.324 (0.714 to 2.456)
CD33 on Im MDSC	Lymphoid leukaemia	6	MR Egger	0.246	1.124 (0.950 to 1.331)
		6	Weighted median	**0.009**	1.089 (1.021 to 1.161)
		6	Inverse variance weighted	**0.024**	1.083 (1.009 to 1.162)
		6	Simple mode	0.258	1.105 (0.947 to 1.290)
		6	Weighted mode	0.058	1.088 (1.016 to 1.165)

S29

exposure	outcome	nsnp	method	pval	OR(95% CI)
HLA DR on monocyte	Androstenediol (3alpha, 17alpha) monosulfate (3)	16	MR Egger	0.310	0.977 (0.934 to 1.021)
		16	Weighted median	0.097	0.972 (0.940 to 1.005)
		16	Inverse variance weighted	**0.041**	0.972 (0.947 to 0.999)
		16	Simple mode	0.450	0.980 (0.932 to 1.031)
		16	Weighted mode	0.126	0.974 (0.943 to 1.006)
Androstenediol (3alpha, 17alpha) monosulfate (3)	Lymphoid leukaemia	7	MR Egger	0.263	1.174 (0.917 to 1.502)
		7	Weighted median	**0.018**	1.185 (1.029 to 1.365)
		7	Inverse variance weighted	**0.014**	1.209 (1.038 to 1.409)
		7	Simple mode	**0.049**	1.449 (1.078 to 1.948)
		7	Weighted mode	0.092	1.175 (1.004 to 1.374)
HLA DR on monocyte	Lymphoid leukaemia	5	MR Egger	0.291	0.819 (0.602 to 1.114)
		5	Weighted median	0.119	0.908 (0.806 to 1.024)
		5	Inverse variance weighted	**0.041**	0.907 (0.825 to 0.996)
		5	Simple mode	0.617	0.954 (0.803 to 1.134)
		5	Weighted mode	0.100	0.869 (0.764 to 0.989)

S30

exposure	outcome	nsnp	method	pval	OR(95% CI)
HLA DR on monocyte	Pregnenetriol disulfate	16	MR Egger	0.798	0.994 (0.949 to 1.041)
		16	Weighted median	0.227	0.975 (0.936 to 1.016)
		16	Inverse variance weighted	**0.044**	0.972 (0.945 to 0.999)
		16	Simple mode	0.522	0.978 (0.916 to 1.045)
		16	Weighted mode	0.933	0.998 (0.955 to 1.043)
Pregnenetriol disulfate	Lymphoid leukaemia	5	MR Egger	0.222	1.323 (0.926 to 1.890)
		5	Weighted median	**0.001**	1.369 (1.134 to 1.652)
		5	Inverse variance weighted	**0.006**	1.334 (1.088 to 1.635)
		5	Simple mode	0.145	1.480 (0.969 to 2.260)
		5	Weighted mode	**0.028**	1.376 (1.142 to 1.657)
HLA DR on monocyte	Lymphoid leukaemia	5	MR Egger	0.291	0.819 (0.602 to 1.114)
		5	Weighted median	0.119	0.908 (0.806 to 1.024)
		5	Inverse variance weighted	**0.041**	0.907 (0.825 to 0.996)
		5	Simple mode	0.617	0.954 (0.803 to 1.134)
		5	Weighted mode	0.100	0.869 (0.764 to 0.989)

S31

exposure	outcome	nsnp	method	pval	OR(95% CI)
HLA DR on B cell	Dihomo−linoleoylcarnitine (C20:2)	21	MR Egger	0.166	1.035 (0.988 to 1.083)
		21	Weighted median	0.052	1.042 (1.000 to 1.086)
		21	Inverse variance weighted	**0.013**	1.038 (1.008 to 1.068)
		21	Simple mode	0.125	1.057 (0.988 to 1.130)
		21	Weighted mode	0.087	1.043 (0.996 to 1.091)
Dihomo−linoleoylcarnitine (C20:2)	Lymphoid leukaemia	4	MR Egger	0.827	1.070 (0.624 to 1.835)
		4	Weighted median	0.124	1.217 (0.948 to 1.560)
		4	Inverse variance weighted	**0.038**	1.264 (1.013 to 1.577)
		4	Simple mode	0.288	1.245 (0.892 to 1.737)
		4	Weighted mode	0.289	1.200 (0.910 to 1.581)
HLA DR on B cell	Lymphoid leukaemia	9	MR Egger	0.304	1.142 (0.903 to 1.445)
		9	Weighted median	**0.036**	1.110 (1.006 to 1.224)
		9	Inverse variance weighted	**0.018**	1.100 (1.017 to 1.189)
		9	Simple mode	0.242	1.076 (0.960 to 1.205)
		9	Weighted mode	0.084	1.091 (1.001 to 1.189)

S32

exposure	outcome	nsnp	method	pval	OR(95% CI)
HLA DR on B cell	1−palmitoyl−2−oleoyl−GPE (16:0/18:1)	21	MR Egger	0.085	0.957 (0.913 to 1.003)
		21	Weighted median	0.144	0.969 (0.929 to 1.011)
		21	Inverse variance weighted	**0.034**	0.968 (0.940 to 0.998)
		21	Simple mode	0.066	0.931 (0.867 to 1.001)
		21	Weighted mode	0.094	0.963 (0.924 to 1.004)
1−palmitoyl−2−oleoyl−GPE (16:0/18:1)	Lymphoid leukaemia	6	MR Egger	0.423	1.206 (0.799 to 1.820)
		6	Weighted median	0.087	1.191 (0.975 to 1.455)
		6	Inverse variance weighted	**0.035**	1.207 (1.014 to 1.437)
		6	Simple mode	0.223	1.262 (0.910 to 1.751)
		6	Weighted mode	0.171	1.185 (0.963 to 1.459)
HLA DR on B cell	Lymphoid leukaemia	9	MR Egger	0.304	1.142 (0.903 to 1.445)
		9	Weighted median	**0.036**	1.110 (1.006 to 1.224)
		9	Inverse variance weighted	**0.018**	1.100 (1.017 to 1.189)
		9	Simple mode	0.242	1.076 (0.960 to 1.205)
		9	Weighted mode	0.084	1.091 (1.001 to 1.189)

Supplement5 | (S1-S32) The causal relationship between immune cell-mediated serum metabolites and Lymphoid leukaemia.
